# PRAP1 is a novel lipid-binding protein that promotes lipid absorption by facilitating MTTP-mediated lipid transport

**DOI:** 10.1074/jbc.RA120.015002

**Published:** 2020-11-24

**Authors:** Hubert Peng, Tzu-Yuan Chiu, Yu-Jen Liang, Chia-Jen Lee, Chih-Syuan Liu, Ching-Shu Suen, Jeffrey J.-Y. Yen, Hung-Ta Chen, Ming-Jing Hwang, M. Mahmood Hussain, Hsin-Chou Yang, Hsin-Fang Yang-Yen

**Affiliations:** 1 Institute of Molecular Biology, Academia Sinica, Taipei, Taiwan; 2 Institute of Statistical Science, Academia Sinica, Taipei, Taiwan; 3 Institute of Biomedical Sciences, Academia Sinica, Taipei, Taiwan; 4 Foundations of Medicine, NYU Long Island School of Medicine, Mineola, New York, USA

**Keywords:** apolipoprotein, lipid absorption, lipid-binding protein, lipoprotein assembly and secretion, lipid transport, mouse, MTTP, PRAP1, triglyceride, apoB, apolipoprotein B, CM, chylomicron, ER, endoplasmic reticulum, HDL, high-density lipoprotein, HFD, high-fat diet, MTTP, microsomal triglyceride transfer protein, PDI, protein disulfide isomerase, PRAP1, proline-rich acidic protein 1, TCHO, total cholesterol, TG, triglyceride, VLDL, very low-density lipoprotein

## Abstract

Microsomal triglyceride transfer protein (MTTP) is an endoplasmic reticulum resident protein that is essential for the assembly and secretion of triglyceride (TG)-rich, apoB-containing lipoproteins. Although the function and structure of mammalian MTTP have been extensively studied, how exactly MTTP transfers lipids to lipid acceptors and whether there are other biomolecules involved in MTTP-mediated lipid transport remain elusive. Here we identify a role in this process for the poorly characterized protein PRAP1. We report that PRAP1 and MTTP are partially colocalized in the endoplasmic reticulum. We observe that PRAP1 directly binds to TG and facilitates MTTP-mediated lipid transfer. A single amino acid mutation at position 85 (E85V) impairs PRAP1's ability to form a ternary complex with TG and MTTP, as well as impairs its ability to facilitate MTTP-mediated apoB-containing lipoprotein assembly and secretion, suggesting that the ternary complex formation is required for PRAP1 to facilitate MTTP-mediated lipid transport. PRAP1 is detectable in chylomicron/VLDL-rich plasma fractions, suggesting that MTTP recognizes PRAP1-bound TG as a cargo and transfers TG along with PRAP1 to lipid acceptors. Both PRAP1-deficient and E85V knock-in mutant mice fed a chow diet manifested an increase in the length of their small intestines, likely to compensate for challenges in absorbing lipid. Interestingly, both genetically modified mice gained significantly less body weight and fat mass when on high-fat diets compared with littermate controls and were prevented from hepatosteatosis. Together, this study provides evidence that PRAP1 plays an important role in MTTP-mediated lipid transport and lipid absorption.

Lipoproteins are macromolecular complexes of various lipids and proteins, and their principal function is to transport lipids. One important class is apolipoprotein B (apoB)-containing lipoproteins that deliver lipids, mainly triglycerides (TGs) and cholesterol, from intestine and liver to other tissues. Two isoforms have been identified for apoB, apoB48 and apoB100, which are encoded by the same gene but generated via a unique mRNA editing process. ApoB100 is synthesized in the liver and is the major structural component of very low-density lipoprotein (VLDL) and its metabolic products. ApoB48, which contains the N-terminal 48% of apoB100, is synthesized in the intestine and is essential for the formation and secretion of chylomicrons (CMs) (1). Unlike human apoB48, the mouse apoB48 isoform is also found in mouse liver (2).

MTTP is an ER resident protein (3, 4). It principally transfers TG to facilitate optimal folding of nascent apoB and also shuttles other lipid classes such as cholesteryl esters, free cholesterol, phospholipids, ceramides, and sphingomyelin to further promote lipoprotein formation (5, 6, 7). MTTP is predominantly expressed in hepatocytes and enterocytes and in several other cell types with relatively less abundance (8, 9). It forms a heterodimer with the ubiquitous ER chaperone protein disulfide isomerase (PDI). PDI itself lacks lipid transfer activity, but its noncovalent association with MTTP significantly promotes the solubility and the lipid transfer activity of MTTP (10). Defective or missing MTTP function is linked to a human disease state, abetalipoproteinemia (11, 12). Mutant mice with conditional intestine-specific deletion of the MTTP gene manifested a significant accumulation of neutral lipids in the villus of the small intestine (13). Both the lipid transfer and apoB-binding properties of MTTP are involved in apoB-lipoprotein assembly (8, 9). Although the crystal structure of human MTTP/PDI complex has been recently reported (14), how exactly MTTP transfers lipids and whether there are other factors involved in MTTP-mediated lipid transport remain elusive.

Proline-rich acidic protein 1 (PRAP1) was initially identified as a protein that was specifically expressed in the uterus during the mid to later stage of pregnancy (15). It was later found that PRAP1 is abundantly expressed in the gut of both humans and mice (https://www.ncbi.nlm.nih.gov/gene/22264; https://www.ncbi.nlm.nih.gov/gene/118471) and is mainly in the small intestinal epithelium with a decreasing expression gradient along the duodenum-ileum axis (16, 17). Overexpression of the human homolog of PRAP1 caused cell growth inhibition in some epithelial and liver cancer cell lines (18). It was also shown that PRAP1 is involved in p53-dependent cell survival after DNA damage (19). However, the physiological and the molecular functions of PRAP1 remain unclear.

We began to be interested in the function of the *PRAP1* gene, because it was pulled out in our screen for genes that were upregulated in uterus shortly after embryo implantation. Here, we demonstrate that PRAP1 is actually a lipid-binding protein that facilitates MTTP-mediated lipid transport.

## Results

### PRAP1-deficient mice manifest increased length of the small intestine

The mouse *Prap1* gene encodes a proline-rich, acidic polypeptide of 149 amino acids (aa) with a putative 20-aa signal peptide. Transient transfection assays using expression vectors encoding PRAP1 C-terminally tagged with HA or FLAG revealed that both tagged molecules were secreted into the culture medium of the transfected cells, suggesting that PRAP1 is a secreted protein (Fig. S1*A*). To delineate the physiological functions of PRAP1, we generated PRAP1 KO mice (PRAP1^−/−^) by a conventional gene-targeting approach (Fig. S1, *B–D*). Homozygous mutants were born with an expected Mendelian frequency and appeared to be normal in their gross appearance (Fig. S1*E*). On a normal chow diet, the body weight increase of the PRAP1^−/−^ mice was quite similar to that of the control mice (Fig. S1*E*). Immunohistochemical analysis of the small intestinal sections from the control mice with PRAP1-specific antibody revealed that the PRAP1 protein was expressed in nearly all epithelial cell lineages (Fig. S2*A*). In the knockout mice, the epithelial-specific expression of PRAP1 was completely ablated (Fig. S2*A*, right panel). Deficiency of PRAP1 did not significantly affect the overall morphology, proliferation, or differentiation in the small intestinal epithelium as revealed by staining the tissue sections with the proliferation marker (Ki-67) (Fig. S3) or markers for various cell lineages (Alcian blue for goblet cells, lysozyme for Paneth cells, and chromogranin A for the enteroendocrine lineage [Fig. S2]). Pulse labeling of mice with bromodeoxyuridine also revealed no significant differences in the migration of intestinal epithelial cells (IECs) between control and PRAP1^−/−^ mice (Fig. S3). However, interestingly, we consistently observed that the length of the small intestine in PRAP1^−/−^ mice was significantly longer than that in control mice (Fig. S1*F*).

### PRAP1 interacts with MTTP and promotes MTTP-mediated lipid transfer and apoB48 lipoprotein secretion

**Figure 1:**
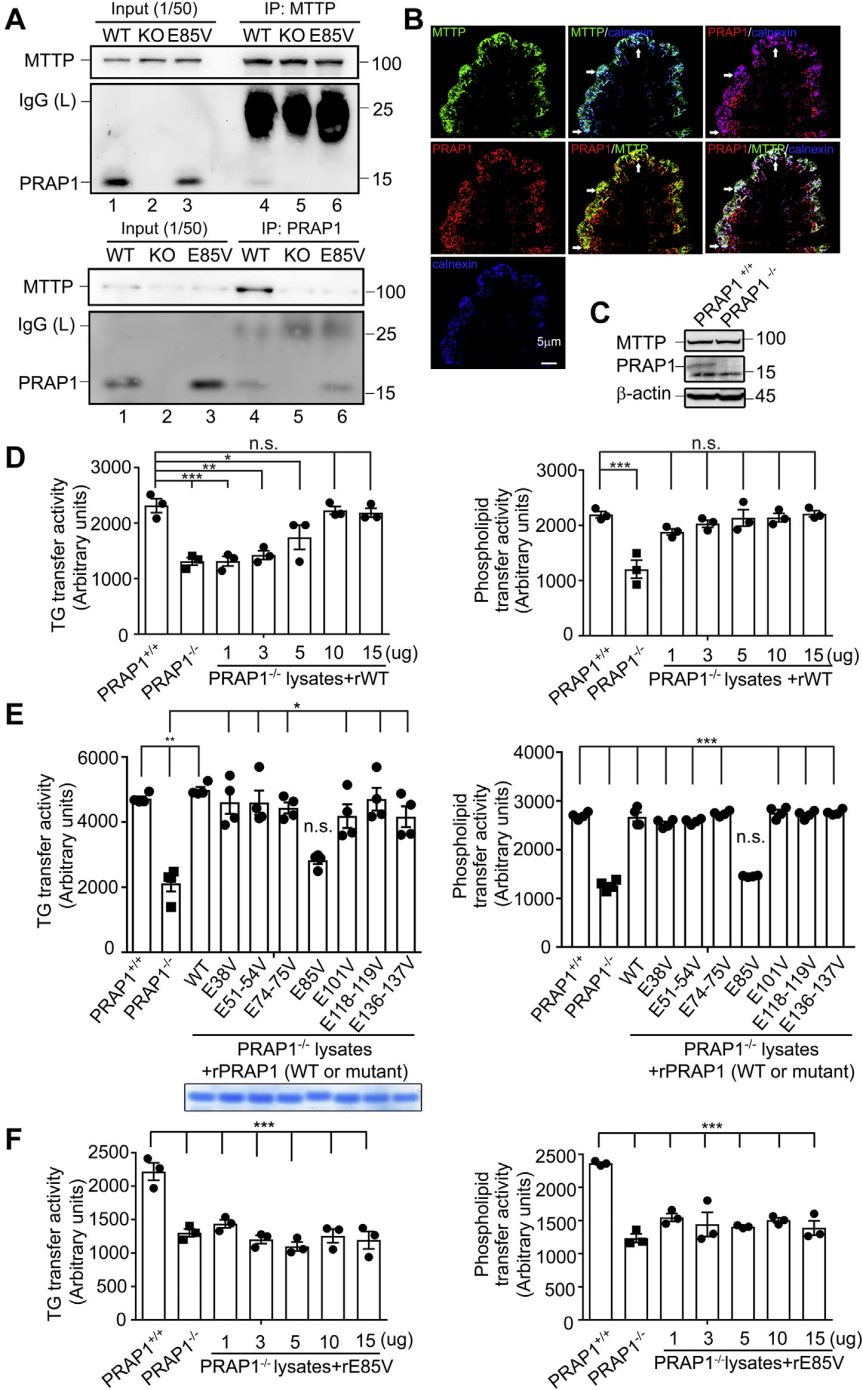
**PRAP1 forms a complex with MTTP and is involved in MTTP-mediated lipid transfer.***A*, total intestinal cell lysates from control (WT), PRAP1^−/−^ (KO), or the E85V mutant mice (E85V) were immunoprecipitated with MTTP- or PRAP1-specific antibody, and the immune complexes (lanes 4–6 in both panels) along with 1/50 input lysates as indicated were analyzed by immunoblotting using MTTP or PRAP1 antibodies. *B*, immunofluorescence staining of MTTP (*green*), PRAP1 (*red*), and the ER marker (calnexin, *blue*) in the small intestinal section of WT mouse. *Arrows* point to examples of colocalized regions. *C*, immunoblotting analysis of MTTP expression in control (WT) and PRAP1^−/−^ (KO) intestinal cell lysates. *D*, recombinant PRAP1 (rPRAP1) stimulated the TG and phospholipid transfer activity of MTTP in the IEC purified from PRAP1^−/−^ mice. MTTP activities in IEC from control or PRAP1^−/−^ mice (bars 1–2) were determined using commercial kits. (Bars 3–7) MTTP-mediated transfer of TG or phospholipids was determined in PRAP1-deficient IEC homogenates that had been preincubated with 1 to 15 μg of WT rPRAP1 (rWT, *i.e.*, PRAP1△N20). *E*, MTTP-mediated transfer of TG or phospholipids was determined in PRAP1-deficent IEC homogenates that had been preincubated with 10 μg of recombinant WT or the indicated mutant of PRAP1 (all without N-terminal 20 aa). The *bottom panel* shows the Coomassie blue staining of the recombinant protein used in that assay. *F*, the E85V mutant of PRAP-1 (rE85V) failed to stimulate the TG or phospholipid transfer activity of MTTP in IEC purified from PRAP1^−/−^ mice at all doses tested. N = 3–4 for each panel. ∗*p* < 0.05; ∗∗*p* < 0.01; ∗∗∗*p* < 0.001; n.s. *p* > 0.05. *D* and *F*, compared with the activity in PRAP1^+/+^. *E*, compared with the activity in PRAP1^−/−^, bar 2.

To explore PRAP1 functions, a pull-down assay using a glutathione-*S*-transferase (GST)-tagged recombinant PRAP1 lacking N-terminal 20-aa signal peptide (GST-PRAP1△N20) and cell lysates prepared from IEC of PRAP1^−/−^ mice was carried out. Mass spectrometry analysis of proteins pulled down by GST-PRAP1△N20 revealed that MTTP is the top candidate that interacts with PRAP1. Further coimmunoprecipitation analysis using either PRAP1 or MTTP-specific antibody confirmed that PRAP1 in IEC from control but not the E85V knock-in mutant mice (see below) could be coimmunoprecipitated with MTTP at the endogenous levels (Fig. 1*A*). Immunostaining confirmed that PRAP1 and MTTP are partially colocalized at the ER (using calnexin as a marker) in the intestinal epithelium (Fig. 1*B*). This result prompted us to examine whether PRAP1 could modulate the lipid transfer activity of MTTP. Figure 1*D* showed that IEC from PRAP1^−/−^ mice manifested significantly reduced TG and phospholipids transfer activity compared with cells from control mice (compare first and second bars), albeit MTTP protein levels in intestinal cells from both genotypes were quite similar (Fig. 1*C*). Interestingly, recombinant (r) PRAP1 lacking the N-terminal signal peptide (*i.e.*, PRAP1△N20, henceforth referred to as WT rPRAP1) could rescue both TG and phospholipids transfer activity of MTTP in PRAP1^−/−^ IEC, where maximal activity could reach to a level that was very similar to that observed using control IEC lysates (Fig. 1*D*). By using such assay, we identified that the Glu residue at position 85 is very important for PRAP1 to promote the lipid transfer function of MTTP, as mutation of such residue to Val (E85V) in PRAP1△N20 markedly attenuated MTTP's activity to transfer both TG and phospholipids *in vitro* (Fig. 1, *E*–*F*). Next, we used HeLa cells that express endogenous PDI to examine the impact of PRAP1 on MTTP-mediated assembly and secretion of apoB48 lipoprotein (20). The results showed that coexpression of PRAP1 indeed enhanced MTTP-mediated secretion of apoB48 into the culture medium (Fig. S4, *A–B*). Consistent with the *in vitro* assay, the E85V mutant of PRAP1 manifested a weaker activity in promoting apoB48 secretion in this cell-based assay (Fig. S4*A*, compare lanes 7–8 to lanes 4–5 and Fig. S4*B*, panels VI *versus* V). Further fractionation of cultured medium by density gradient ultracentrifugation (21) revealed that, in this transient expression system, apoB48 was secreted into the HDL-sized fraction when PRAP1 was coexpressed with apoB48 in the same cells (Fig. S4*B*, panel labeled “V”).

### PRAP1 binds to TG and interacts with MTTP as a ternary complex

**Figure 2:**
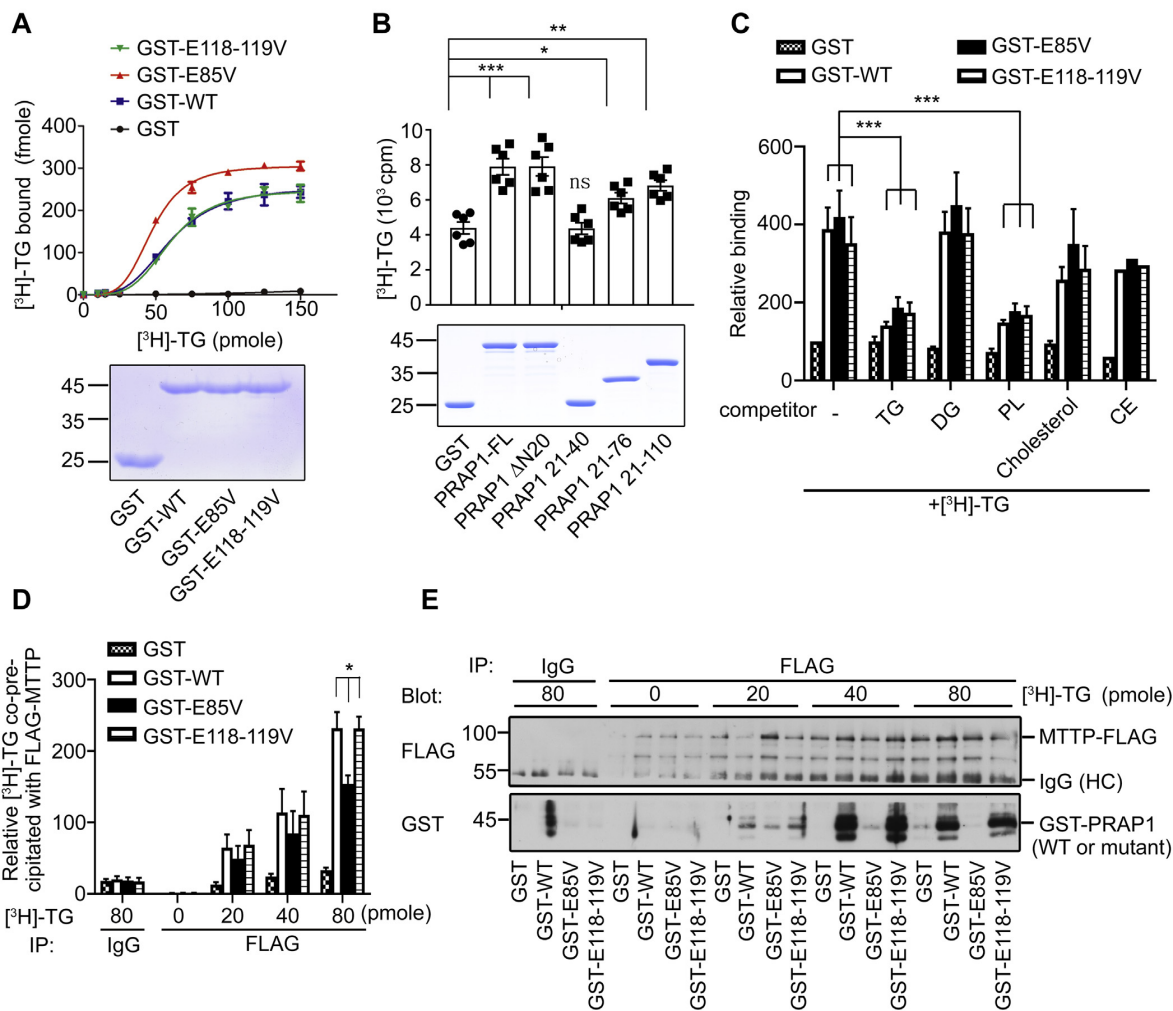
**PRAP1 directly binds to TG and forms a ternary complex with MTTP.***A*, lipid (^3^H-labeled TG, [^3^H]-TG) binding curves of bacterially produced GST or GST fusion proteins containing WT or the indicated mutant of PRAP1 (∼0.7 μg each, Coomassie blue stained, *bottom panel*). *B*, lipid-binding ability of GST-fusion proteins containing WT or the truncated forms of PRAP1 (∼0.7 μg each, *bottom panel*). *C*, PRAP1 also binds to phospholipids. Approximately 0.7 μg of the indicated proteins were incubated with 40 pmole of [^3^H]-TG in the absence or presence of the indicated excess unlabeled lipids (10 nM). Data are presented as mean ± SEM, and the results are averages from three independent experiments except for competition with cholesterol oleate, which was done once in triplicate. All binding activity was normalized to those bound to GST in the absence of any competitor (bar 1), which was set as 100. *D* and *E*, FLAG-tagged MTTP immunoprecipitated from HeLa cells was allowed to bind to recombinant WT or the indicated mutant protein preloaded without or with increasing doses of [^3^H]-TG. After incubation for 4 h, the immune complex was washed and eluted, and half of the sample was subjected to isotope counting for [^3^H]-TG (*D*), and the other half was used for immunoblotting analysis using FLAG or GST antibody as indicated (*E*). ∗*p* < 0.05; ∗∗*p* < 0.01; ∗∗∗*p* < 0.001; n.s. *p* > 0.05. Note, except PRAP1-FL (*B*), all recombinant PRAP1 (wt or mutant) used here are without N-terminal signal peptides.

Next, we tested the hypothesis that PRAP1 is a lipid-binding protein. Figure 2 shows that GST-PRAP1△N20 (labeled as GST-WT in the figure) indeed bound to ^3^H-labeled TG in a saturable manner (Fig. 2*A*) and the region between aa residues 40 to its C-terminal end appeared to be important for TG binding (Fig. 2*B*). Of note, the presence of the signal peptide in the full-length recombinant protein (labeled as PRAP1-FL) did not further increase the lipid binding activity of PRAP1△N20 (compare PRAP1-FL and PRAP1△N20 in Fig. 2*B*). Interestingly, the PRAP1 mutant, which retains wt-like activity (the E118-119V mutant) in promoting MTTP-mediated lipid transfer, manifested a similar lipid-binding property as the WT protein, whereas the E85V mutant manifested a distinct property where its binding capacity to TG was markedly increased compared with the WT or the E118-119V mutant (Fig. 2*A*). Further competition experiments using excess unlabeled lipids revealed that phospholipids could significantly compete with TG for binding to PRAP1 (Fig. 2*C*), suggesting that the binding sites of these two lipids to PRAP1 significantly overlap with each other. Of note, no significant or marginal competition could be observed for diacylglycerides, cholesterol, or cholesteryl oleate in this assay (Fig. 2*C*). The E85V mutant binds to TG with a higher capacity than the WT control (compare GST-E85V and GST-WT in Fig. 2*A*), but it fails to promote the lipid transfer activity of MTTP (Fig. 1). These results suggest a possibility that MTTP may recognize PRAP1-bound TG as a cargo and then transfer TG to the lipid acceptor like apoB and that the E85V-bound lipids are not as accessible to MTTP as the WT protein-bound lipids. If such model is correct, one would expect to detect a ternary complex containing MTTP and the lipid-loaded PRAP1, but the E85V mutation would significantly impair the formation of such ternary complex. To test this hypothesis, FLAG-tagged MTTP immunoprecipitated from HeLa cells was allowed to bind to recombinant WT (GST-WT) or the E85V mutant of PRAP1 (GST-E85V) preloaded without or with increasing doses of ^3^H-labeled TG. The E118-119V mutant of PRAP1, which behaved like WT protein in various assays mentioned above (Fig. 1 and Fig. 2, *A* and C), was included in this assay. Figure 2*D* shows that significantly more ^3^H-labeled TG was coprecipitated with FLAG-tagged MTTP in a binding reaction containing GST-WT or GST-E118-119V than in a reaction containing GST-E85V. Moreover, significantly more GST-WT or GST-E118-119V was coprecipitated with FLAG-tagged MTTP than GST-E85V in the presence (40–80 pmole of ^3^H-TG under our assay conditions) but not in the absence of lipids (Fig. 2*E*). Consistent with this result, MTTP antibody could coimmunoprecipitate endogenous WT but not the E85V mutant of PRAP1 in IEC lysates (Fig. 1*A*). Together, these results support a model that PRAP1 interacts with TG, which renders TG to be more accessible to MTTP during its lipid transfer reaction.

### PRAP1 facilitates lipid absorption and apoB-lipoprotein assembly and secretion in mice

**Figure 3:**
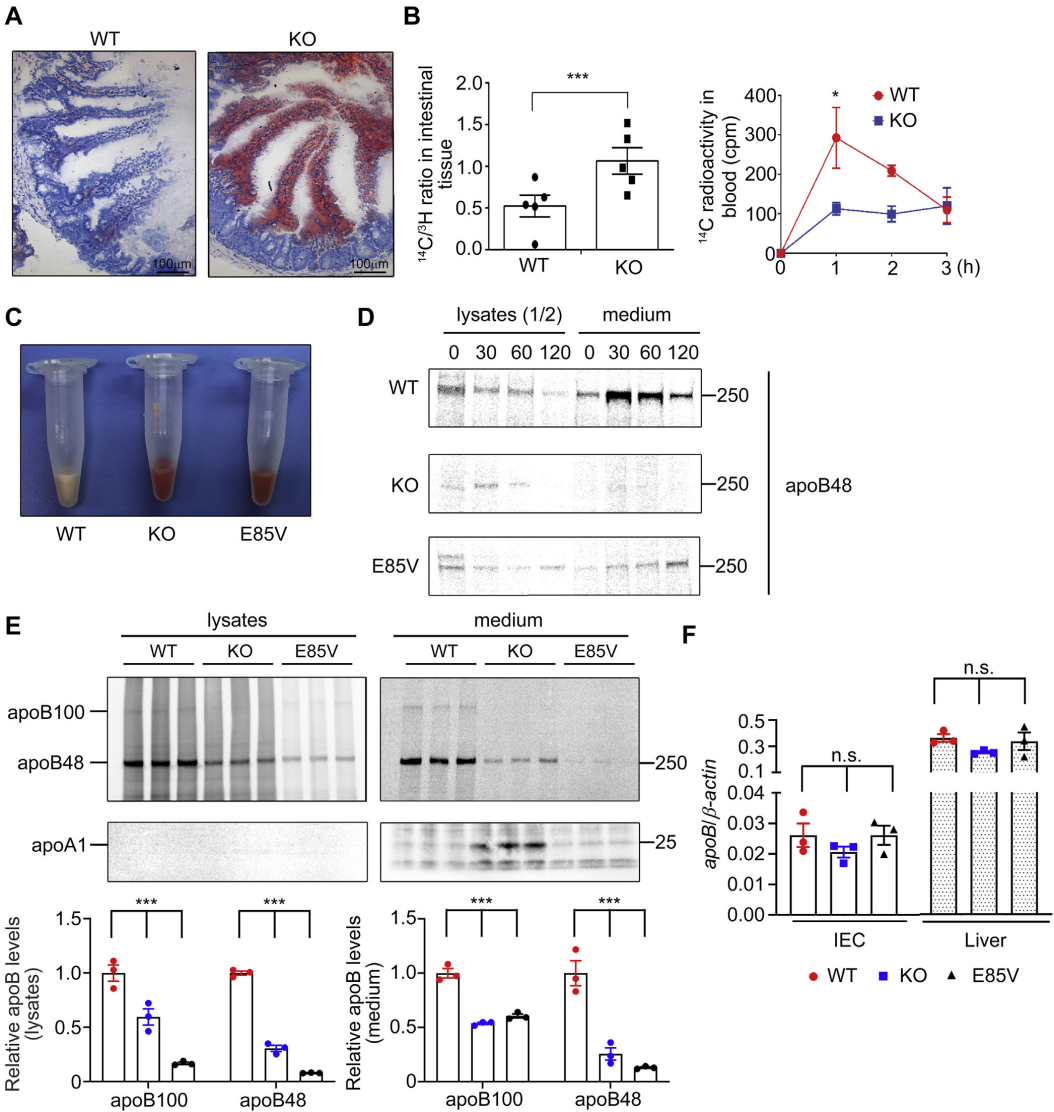
**PRAP1 facilitates lipid absorption and apoB lipoprotein assembly and secretion in mice.***A*, mice with the indicated genotype were deprived of food for 12 h before receiving an intragastric bolus of lipid. Two hours later, small intestinal segments were processed for staining by Oil red O. *B*, reduced lipid absorption in the small intestine of PRAP1^−/−^ mice. Control or PRAP1^−/−^ mice (n = 5 each) were deprived of food for 12 h before receiving an intragastric bolus of 0.5 ml of corn oil containing 5 μCi of [^14^C]-Triolein and 1 μCi of [^3^H]-β-sitostanol. Two hours (left) or 1 to 3 h (right) after lipid administration, the amount of [^14^C]-Triolein-derived radioactivity in the cell lysates from the middle part of the small intestine (as a ratio of [^14^C]/[^3^H] radioactivity, left panel) or in the blood sample (right panel) was measured and plotted. ∗*p* < 0.05; ∗∗∗*p* < 0.001. *C*, mice with the indicated genotypes were fasted for 16 h before receiving oral gavage of lipids. Four hours post lipid administration, plasma was collected and photographed. *D* and *E*, PRAP1 deficiency or the E85V mutation reduces apoB lipoprotein secretion. *D*, pulse chase experiments using primary enterocytes isolated from mice with the indicated genotypes were carried out and ^35^S-labeled apoB was immunoprecipitated from media and lysates were subjected to SDS-PAGE and fluorography. One half of the immunoprecipitated apoB from cell lysates was loaded in this gel. *E*, metabolic labeling experiments using primary hepatocytes isolated from mice with the indicated genotypes were performed and ^35^S-labeled apoB and apoA1 sequentially immunoprecipitated from media and lysates were subjected to SDS-PAGE and fluorography. Note: the images shown for the lysates and the medium were separately adjusted for their brightness and contrast, thus not reflecting ∼3 times higher signals in medium than in the lysates. The bottom panel shows the quantification of apoB signals in the upper panel. All signals were normalized to the mean of those triplicates in WT cells. *F*, ApoB mRNA levels in purified IEC or liver from mice with the indicated genotypes were analyzed by RT-qPCR. Each data point denotes an individual mouse, and the bars denote mean ± SEM. n.s., *p* > 0.05.

**Figure 4:**
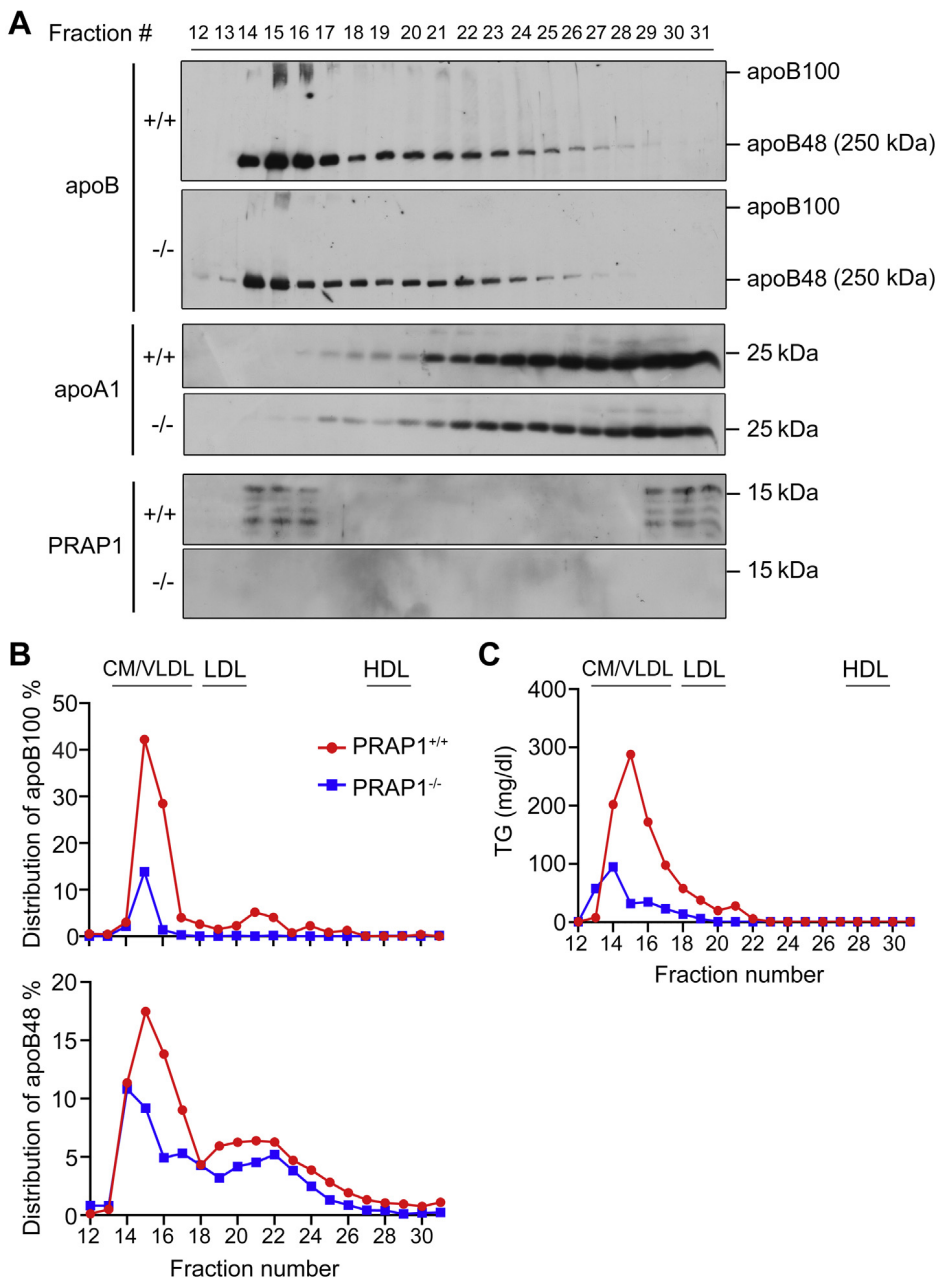
**PRAP1 deficiency reduces apoB-lipoprotein assembly and secretion into the chylomicron/very low-density lipoprotein–rich fractions.** Control (^+/+^) or PRAP1^−/−^ mice were fasted for 16 h before tyloxapol injection and receiving an oral lipid bolus. Four hours later, the plasma pooled from three mice of the same genotype was fractionated by fast phase liquid chromatography, followed by immunoblotting analysis of the indicated protein (*A*) or measurement of triglyceride (TG) levels (*C*) in each fraction. The apoB protein signal in each fraction was quantitated and plotted as a relative percentage of total apoB from all 20 fractions from WT plasma (*B*).

**Figure 5:**
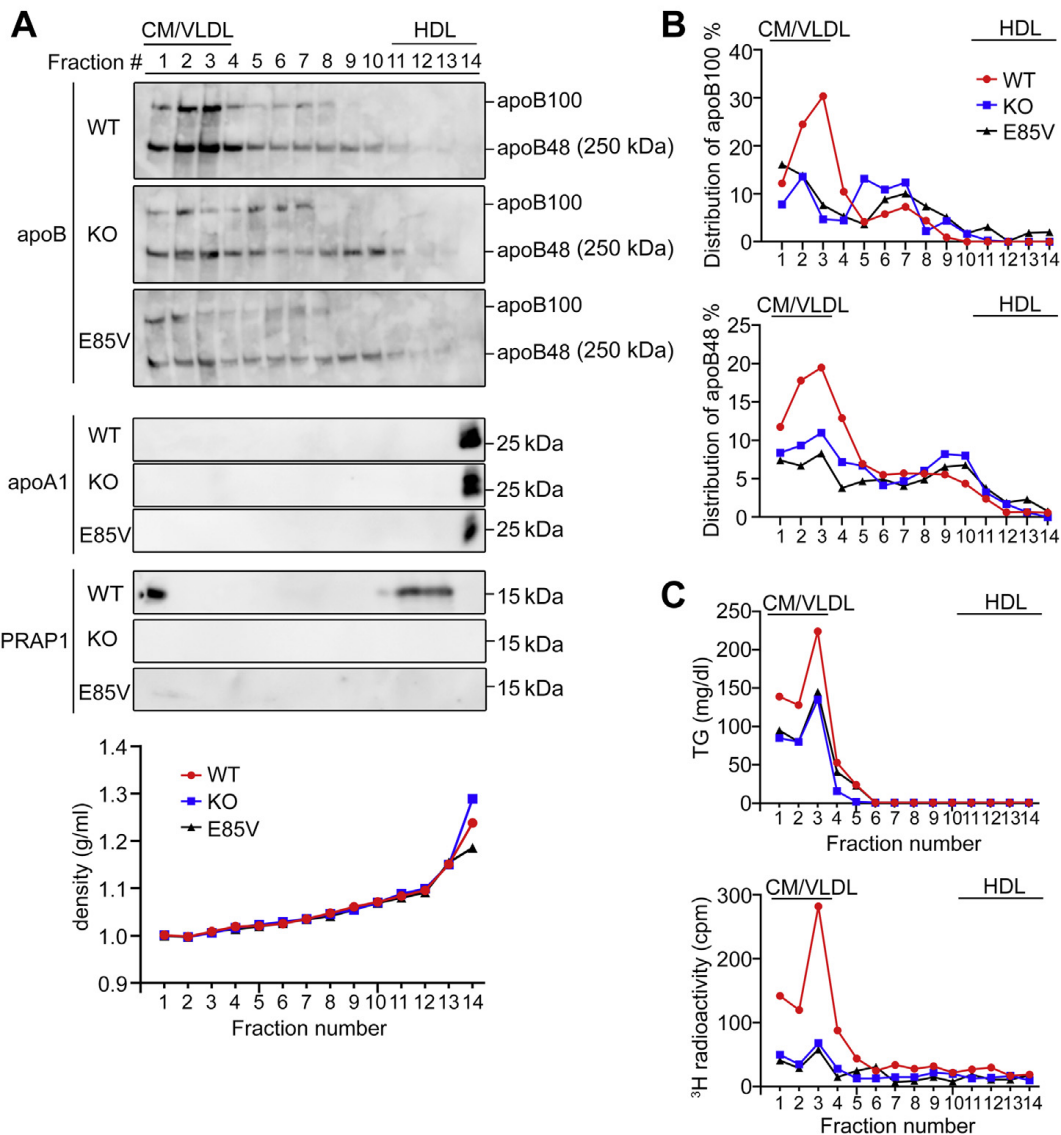
**The E85V mutation reduces apoB-lipoprotein assembly and secretion into the chylomicron/very low-density lipoprotein–rich fractions.** Mice with the indicated genotypes were fasted for 16 h before tyloxapol injection and receiving an oral lipid bolus containing 10 μCi of [^3^H] triolein. Three hours later, plasma (600 μl) pooled from three mice of the same genotype was fractionated by density gradient ultracentrifugation, followed by analysis of the indicated proteins by immunoblotting (*A*). *B*, the apoB protein signal in each fraction shown in (*A*) was quantitated and plotted as a relative percentage of total apoB from all 14 fractions from WT plasma. *C*, triglyceride (TG) mass or [^3^H] radioactivity in each fraction analyzed in (*A*). Shown here is one representative result from two independent experiments. See also Figure S7 for total apoB and TG levels of each individual mouse plasma analyzed in (*A–C*).

We next examined whether PRAP1 is involved in lipid absorption in mice. Figure 3 shows that, 2 h following oral administration of a bolus of lipid, significantly more lipid (revealed by Oil red O staining, Fig. 3*A*, or by measurement of [^14^C]-Triolein-derived radioactivity, Fig. 3*B*) was detected in the small intestinal tissue of the PRAP1^−/−^ mice compared with that in the control mice, whereas the level of [^14^C]-Triolein-derived radioactivity detectable in the bloodstream was much less in PRAP1^−/−^ mice than in the control mice (Fig. 3*B*, right panel). Consistent with this result, the plasma collected from PRAP1^−/−^ mice 4 h post lipid administration was not as creamy white as that from control mice (Fig. 3*C*), suggesting that PRAP1 deficiency decreases lipid absorption. Next, since MTTP is essential for the biosynthesis of apoB-containing lipoproteins (22), we examined whether PRAP1 would affect apoB-lipoprotein assembly and secretion from two major apoB-producing cell types. Pulse chase and radiolabeling experiments revealed that the synthesis and secretion of newly synthesized apoB48 was significantly reduced from primary IEC purified from PRAP1^−/−^ or the E85V mutant mice compared with that from the control mice (Fig. 3*D*), albeit PRAP1 deficiency or the E85V mutation did not significantly affect apoB mRNA expression (Fig. 3*F*). Similar results were observed in experiments using primary hepatocytes where significantly less apoB48 and apoB100 were synthesized and secreted from both PRAP1^−/−^ and the E85V mutant hepatocytes compared with control cells (Fig. 3*E*). Next, we examined the amount and lipidation of apoB in plasma of mice that had been fasted for 16 h before they were intravenously injected with tyloxapol (a lipoprotein lipase inhibitor) and received an intragastric bolus of lipid emulsion containing Intralipid and corn oil. Fractionation of plasma from 4 h post-lipid-administered mice using fast phase liquid chromatography (23) revealed that significantly less apoB48 and apoB100 and TG were distributed to the CM/VLDL-rich fraction of PRAP1^−/−^ plasma (fractions 14–16, Fig. 4), compared with the same fraction from control mouse plasma (Fig. 4). Further fractionation of plasma from 16-h-fasted mice by density gradient ultracentrifugation revealed that PRAP1 deficiency slightly reduced apoB lipidation and secretion into hepatic VLDL (Fig. S5), suggesting that a very low level of PRAP1 in the liver (compared with that in IEC, see Fig. S6) still exerts some effects on MTTP-mediated apoB lipoprotein assembly and secretion. Additional similar fractionation experiments using plasma from 3 h post-lipid-administered mice showed that the E85V mutant mice manifested very similar phenotype as the PRAP1^−/−^ mice, *i.e.*, significantly less apoB48 and apoB100 and TG were distributed into the CM/VLDL-rich fractions (fractions #1–3) of PRAP1^−/−^ and E85V mutant plasma compared with those of control plasma (Fig. 5 and Fig. S7).

Of note, in the experiment shown in Figures 4 and 5, PRAP1 was readily detectable in the plasma of 3–4 h post-lipid-administered mice. A basal level could be detected at the 0-h time point (after 4-h fast) if more plasma was analyzed for the immunoblotting analysis (Fig. S8*B*). Interestingly, although the E85V mutant protein could be readily detected in the intestinal epithelium by immunohistochemical analysis (Fig. S8*A*), it was less secreted into bloodstream or secreted with a slower secretion kinetics compared with the WT protein (Fig. S8*B*). Notably, PRAP1 was frequently detected as multiple bands (Fig. 4 and Fig. S4*A*). The identity of these bands remains to be determined.

### Both PRAP1^−/−^ and the E85V mutant mice gain less body weight and fat mass and are prevented from hepatosteatosis on high-fat diets

**Figure 6:**
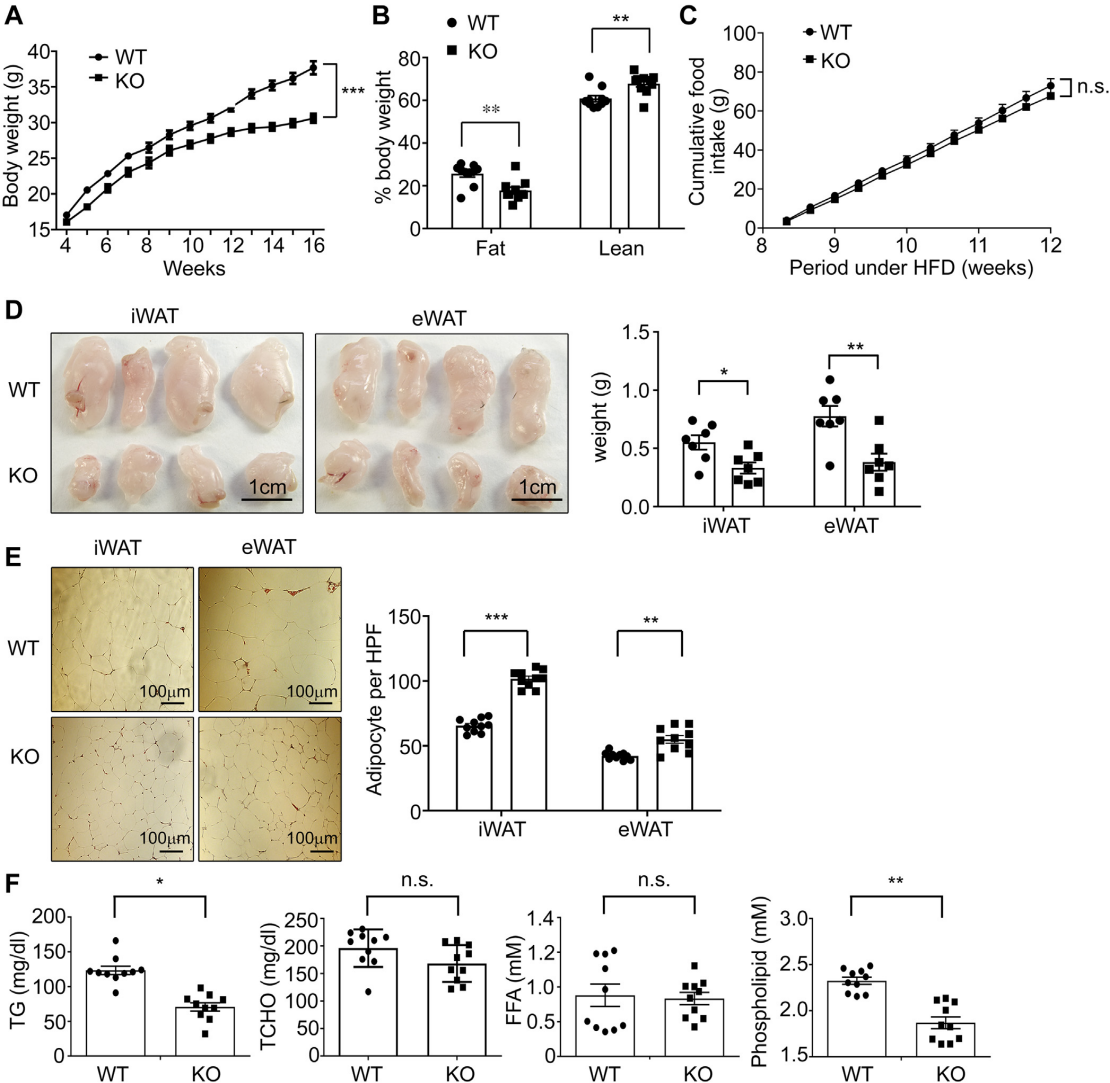
**PRAP1-deficient mice gained less body weight and fat mass on a HFD.***A*, body weight of control (WT) or PRAP1^−/−^ (KO) mice fed a HFD for 12 weeks was measured and plotted against time. N = 10 for each genotype. *B*, time domain NMR analysis of the lean and fat mass of mice analyzed in (*A*) at the end of the experiment. *C*, cumulative food intake of the indicated mice measured from week 8 to 12 under HFD. N= 5 for WT; N = 4 for KO. *D*, left panel, representative images of iWAT and eWAT from mice analyzed in (*A*); right panel shows the average weight of the indicated tissues. *E*, representative H&E-stained sections of the indicated tissues. Right panel shows average adipocyte numbers per high power field (HPF). *F*, plasma levels of TG, phospholipids, total cholesterol (TCHO), or free fatty acids (FFA) of mice analyzed in (*A*) after 12 weeks on a HFD. Each data point denotes an individual mouse, and the bars denote mean ± SEM. ∗*p* < 0.05; ∗∗*p* < 0.01; ∗∗∗*p* < 0.001, n.s., *p* > 0.05.

**Figure 7:**
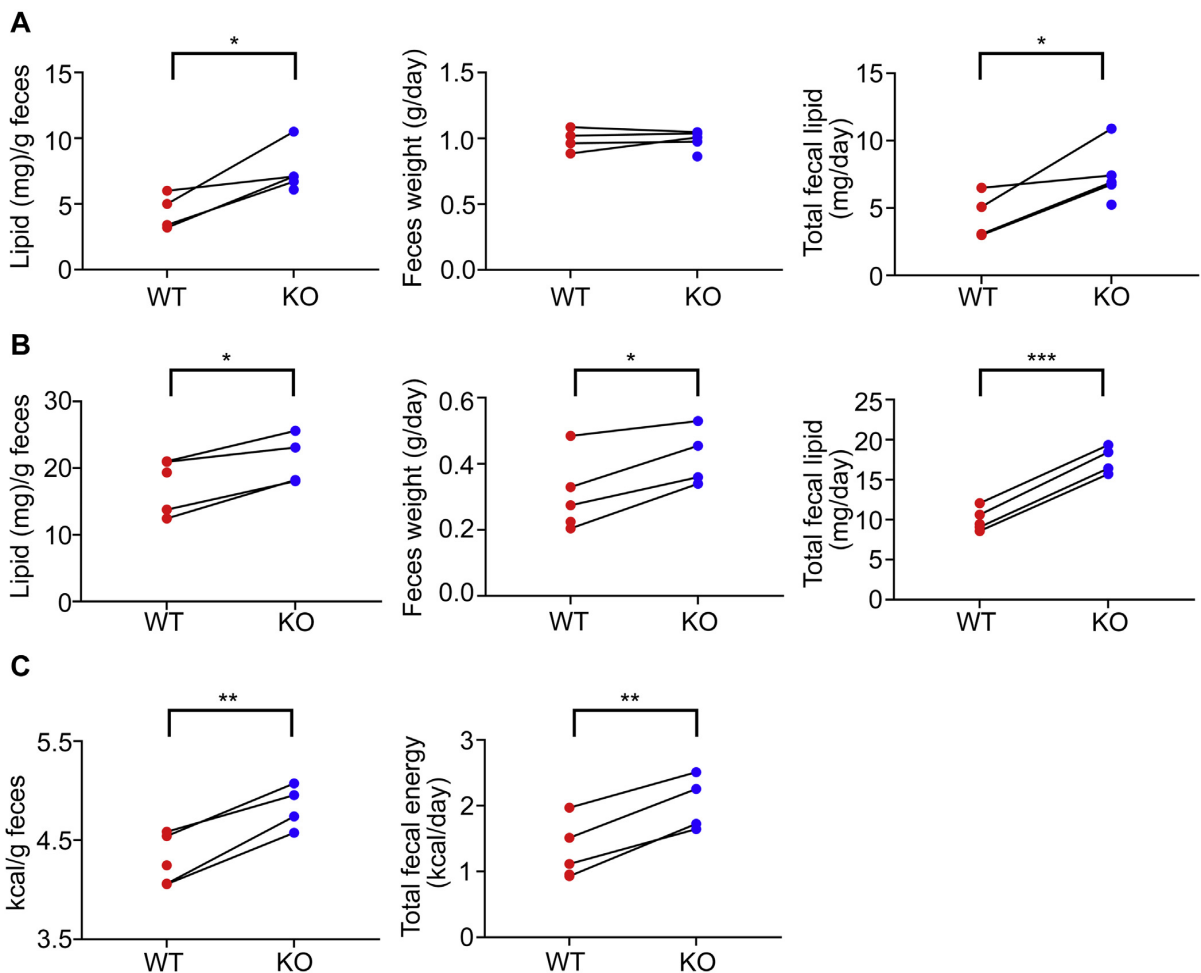
**PRAP1**^**−/−**^**mice had increased fecal lipid content and absorbed less calories from the diet.***A*, fecal lipid content of individually housed WT and PRAP1^−/−^ mice fed with a chow diet. Feces over four consecutive days were collected from 14- to 16-week-old mice and dried. Fecal lipid content was then determined after chloroform/methanol extraction. *B*, individually housed WT and PRAP1^−/−^ mice at 4 weeks of age were fed with a high-fat diet for 2 weeks. After that, feces collected from two consecutive days were dried, and the lipid content was determined after chloroform/methanol extraction. *C*, a portion of feces collected from mice described in (*B*) was homogenized and subjected to analysis in triplicates using Parr 6725 semimicro oxygen bomb calorimeter. Energy content per gram of feces and total fecal energy per day are plotted. Data are cumulative results from three independent experiments, each with 1 to 2 WT or KO mice from the same litter. ∗*p* < 0.05; ∗∗*p* < 0.01; ∗∗∗*p* < 0.005; paired Student's *t* test.

Although on chow diet the body weight increase of control and knockout mice were quite similar (Fig. S1*E*), on high-fat diet (HFD) (58Y1-60% energy from fat, Test Diet) PRAP1^−/−^ mice gained significantly less body weight compared with control mice (Fig. 6*A*). Time domain -NMR analysis confirmed that, after 12 weeks on a HFD, the fat mass percentage of the knockout mice was significantly reduced compared with that of the control mice (Fig. 6*B*). Consistent with this result, both adipose tissues examined (iWAT, eWAT) were significantly smaller in size in PRAP1^−/−^ mice compared with that in the corresponding tissue in the control mice (Fig. 6*D*), and PRAP1^−/−^ adipocytes were significantly smaller than the control cells (Fig. 6*E*). Given that both control and PRAP1^−/−^ mice had very similar food intake on a HFD (Fig. 6*C*), we next compared their energy expenditure and fecal lipid content. Both groups of mice manifested very similar energy expenditure as revealed by the Comprehensive Laboratory Animal Monitoring System (Fig. S9). However, feces of PRAP1^−/−^ mice contained more lipids than feces of control mice (Fig. 7, *A*–*B*). Bomb calorimetry also revealed that more calories were retained in PRAP1^−/−^ feces than in control feces (Fig. 7*C*), suggesting that the leaner phenotype of PRAP1^−/−^ mice on a HFD was mainly due to a defect in lipid absorption. Last, after 12 weeks on a HFD, a significant decrease in the steady state (with *ad libitum* access to food and water) plasma level of TG and phospholipids was observed in PRAP1^−/−^ mice (Fig. 6*F*), albeit total cholesterol (TCHO) or free fatty acid levels in the plasma from both groups of mice appeared to be very similar (Fig. 6*F*).

**Figure 8:**
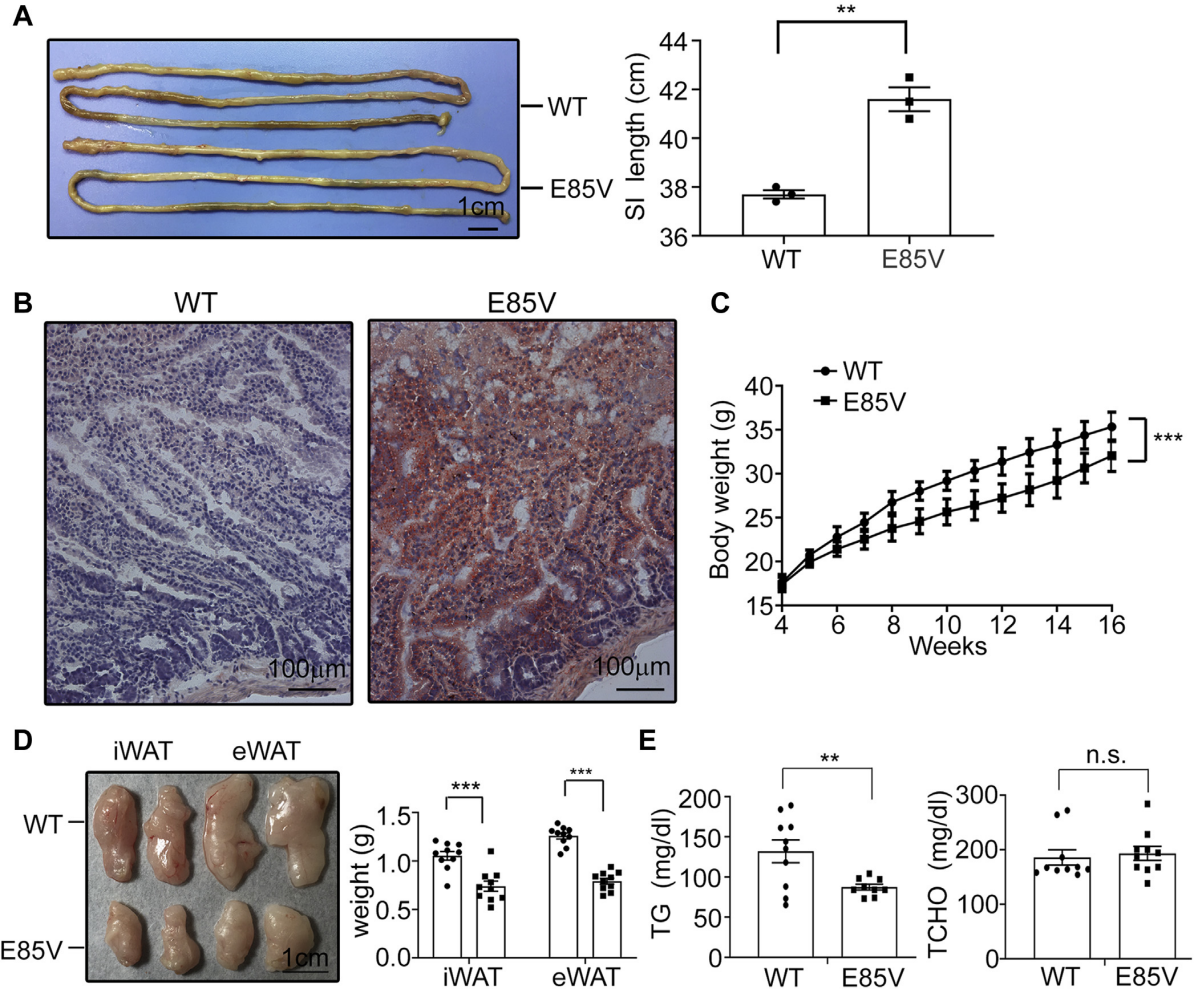
**The E85V mutant mice gained less body weight and fat mass on high-fat diet (HFD).***A*, the small intestine of chow diet–fed E85V mutant mice is longer than that of control mice. Shown here is one representative photo from three mice with each indicated genotype. *B*, mice with the indicated genotype were deprived of food for 12 h before receiving an intragastric bolus of lipid. Two hours later small intestinal segments were processed for staining by Oil red O. *C*, body weight of control or E85V mice fed a HFD for 12 weeks was measured and plotted against time. n = 10 for each genotype. *D*, left panel, representative images of iWAT and eWAT from mice analyzed in (*C*); right panel shows the average weight of the indicated tissues. *E*, plasma levels of triglyceride (TG) and total cholesterol (TCHO) of mice analyzed in (*C*) after 12 weeks on HFD. Each data point denotes an individual mouse, and the bars denote mean ± SEM. ∗∗*p* < 0.01; ∗∗∗*p* < 0.001, n.s., *p* > 0.05.

**Figure 9:**
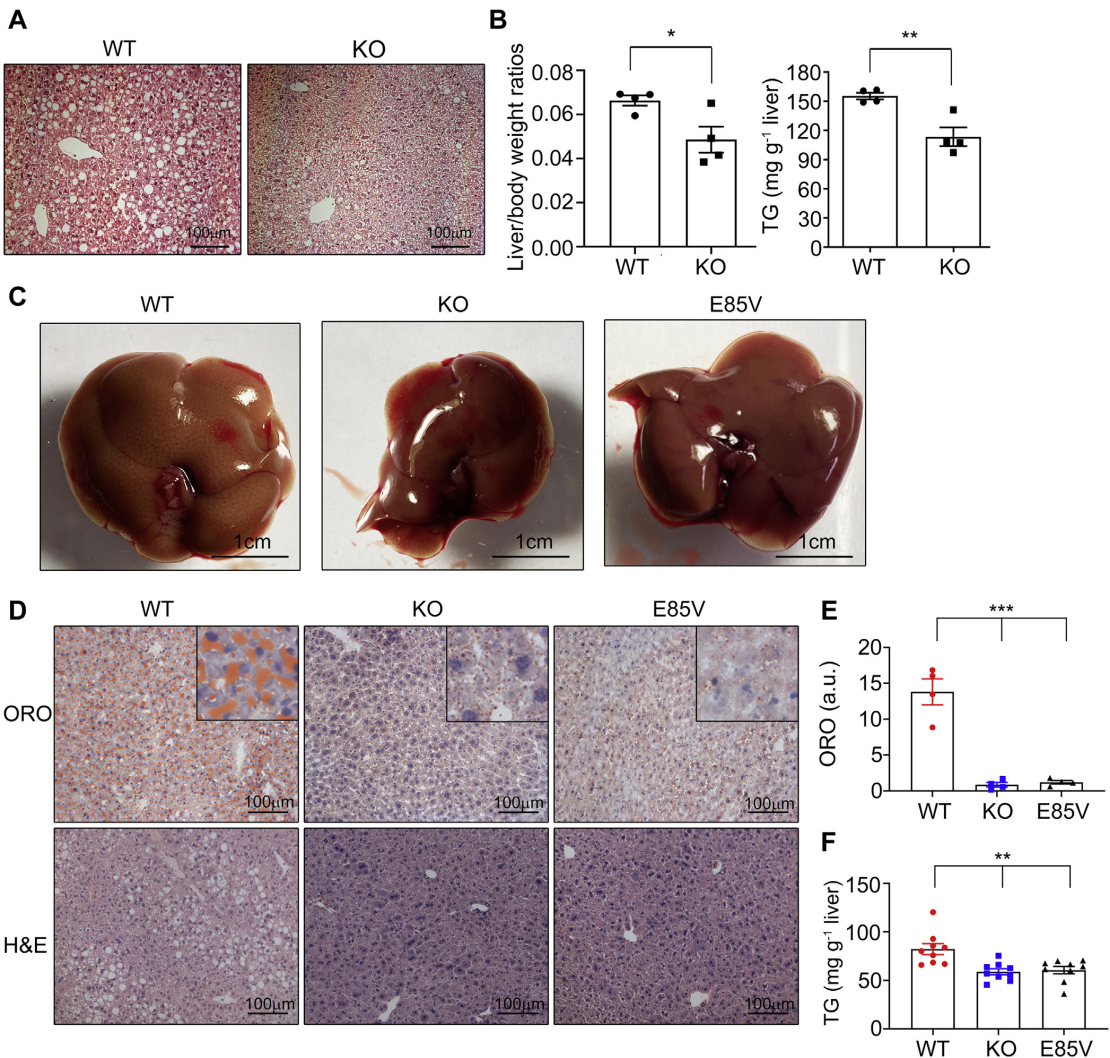
**PRAP1 deficiency or the E85V mutation diminishes high-fat diet (HFD)-induced hepatosteatosis.***A* and *B*, control (WT) and PRAP1^−/−^ (KO) mice (n = 4, each) were fed with a HFD for 12 weeks. Representative H&E-stained sections of livers from these mice are shown in (*A*) and their liver/body weight ratios and liver triglyceride (TG) contents are shown in (*B*). *C–F*, mice with the indicated genotypes were fed with a HFD for 8 weeks. Representative gross appearance of the livers (*C*), H&E- and Oil Red O–stained liver sections (*D*) from these mice are as indicated. *E*, quantification of lipid droplets shown in (*D*) by the ImageJ software. *F*, liver TG contents of mice fed with a HFD for 8 weeks. Each data point denotes an individual mouse, and the bars denote mean ± SEM. ∗*p* < 0.05; ∗∗*p* < 0.01; ∗∗∗*p* < 0.001. N = 4–9.

Interestingly, the E85V mutant mice manifested very similar phenotypes as the knockout mice in many aspects. For examples, their small intestine was consistently longer than that of littermate controls (Fig. 8*A*, fed on a chow diet). More lipids were accumulated in the small intestinal tissues of the E85V mutants compared with control mice after oral gavage of lipids (Fig. 8*B*). In addition, on HFD for 12 weeks, compared with control mice, the E85V mutant mice gained significantly less body weight (Fig. 8*C*) and manifested smaller adipose tissues (iWAT and eWAT) (Fig. 8*D*) and lower plasma TG levels (Fig. 8*E*). Last, we examined the impact of PRAP1 deficiency or the E85V mutation on HFD-induced lipid accumulation in the liver (hepatosteatosis). For this experiment, mice were fed a HFD for 12 (Fig. 9, *A*–*B*) or 8 weeks (Fig. 9, *C*–*F*) before their livers were processed and analyzed for morphology and lipid contents. The results show that lipid droplets (stained by Oil red O, Figure 9, *D*–*E*), hepatocytes with vacuolation (by H&E staining, Fig. 9, *A* and *D*), and hepatic TG contents (Fig. 9, *B* and *F*) were all significantly reduced in PRAP1^−/−^ and the E85V mutant livers compared with controls, suggesting that PRAP1 deficiency or the E85V mutation diminishes HFD-induced hepatosteatosis.

## Discussion

Here, we provide evidence that PRAP1 is a novel player in MTTP-mediated transfer of lipids (mainly TG and phospholipids). Our results indicate that the ability to form a ternary complex with TG and MTTP correlates with the ability of PRAP1 to promote MTTP-mediated lipid transfer. Earlier studies suggest that MTTP binds and shuttles lipid molecules between membranes and such shuttle mechanism for lipid transport predicts that a stable MTTP-lipid complex is an intermediate in the transfer reaction (3, 24, 25). Our results suggest that PRAP1 may be the key molecule that promotes the formation of the stable MTTP–lipid complex predicted in these earlier studies. Moreover, this study strongly supports the possibility that MTTP recognizes PRAP1-bound TG as a cargo and then transfers TG along with PRAP1 to the lipid acceptors apoB, rendering PRAP1 to be one component of chylomicrons/VLDL. Given that PRAP1 is a secreted protein and is partially colocalized with MTTP at ER, we propose a model that, during its secretion process, PRAP1 may encounter TG produced in the ER membrane and then acts like a detergent to help solubilize TG and present it to MTTP. More experiments would be required to test this model. Moreover, this study identified an interesting, loss-of-function mutant of PRAP1 (the E85V mutation) that cannot promote the lipid-transfer activity of MTTP. The E85V mutant binds to TG but fails to form a ternary complex with TG and MTTP. It is expressed, but is much less secreted or secreted with a much slower kinetics (Fig. S8). This mutation causes a phenotype that is very similar to that caused by PRAP1 deficiency in terms of apoB secretion and lipid absorption. Based on these properties, we propose that E85V-bound lipids may not be as “soluble” as the WT protein-bound lipids and thus are less accessible to MTTP. Alternatively, MTTP may only recognize WT protein- but not E85V mutant-bound lipids, owing to differences in the conformation of these two protein–lipid complexes. Further structural studies would be required to address this issue.

PRAP1 deficiency reduces lipid absorption. We show that apoB can still be secreted in PRAP1^−/−^ mice but is significantly less in quantity and in the extent of lipidation. The increased length of the small intestine of PRAP1^−/−^ mice compared with control mice on the same chow diet suggests that this is a compensatory response for the animal to absorb more lipids. MTTP knockout mice die early during embryonic development, whereas the Mttp^+/−^ mice with half-normal levels of MTTP gene expression are viable, albeit with decreased secretion of apoB and reduced plasma apoB levels (26). The phenotype of Mttp^+/−^ mice may partially explain why PRAP1^−/−^ mice are viable. MTTP is mainly expressed in hepatocytes and enterocytes. Conditional knockout of MTTP in hepatocytes caused moderate hepatic steatosis (27). However, hepatic TG content was significantly reduced in IEC-specific MTTP knockout mice, which is consistent with decreased intestinal lipid delivery (13). PRAP1 deficiency diminishes HFD-induced hepatosteatosis, suggesting that PRAP1 dominates its role in facilitating MTTP activity in IEC than in hepatocytes, at least in mice. Consistent with this notion, PRAP1 expression is found to be much lower in mouse livers than in IEC (Fig. S6).

Current models suggest that the assembly of apoB-lipoprotein is likely a two-step process. The first step is the transfer of a small amount of lipid to nascent apoB during its translocation into the ER lumen to form a primordial apoB particle. The second step “core expansion” has been suggested to exist, *i.e.*, the fusion of the HDL-sized primordial apoB particles with preformed ER luminal lipid droplets that do not contain apoB (9, 28, 29, 30). MTTP is required for the formation of primordial apoB particles and ER luminal lipid droplets (13, 27, 31, 32). Whether the formation of the PRAP1-TG-MTTP complex is involved in both steps of apoB-lipoprotein assembly remains to be determined. On the other hand, during the fractionation of plasma, we noticed that PRAP1 is also detectable in lipid-poor fractions, *i.e.*, HDL. The origin and function of PRAP1 in these lipid-poor fractions remain to be determined.

Plasma levels of TCHO, low-density lipoprotein cholesterol, high-density lipoprotein cholesterol, and TG are among the most important risk factors for coronary artery disease. Two large-scale genome-wide association studies (GWASs) have revealed many loci significantly associated with lipid traits (33, 34). Analysis of datasets from these two studies, as well as several other databases including GWAS Central (35), GWAS Catalog (36), Phenotype-Genotype Integrator (PheGenI) (37), Gene Expression Database (GEO) (38), Online Mendelian Inheritance in Man (OMIM) (39), and Open Targets (40) revealed no significant genetic association between published single nucleotide polymorphisms (SNPs) on PRAP1 or PRAP1 gene expression and lipoprotein-related traits. This negative result, however, does not exclude the possibility that other yet to be identified SNPs on PRAP1 might be associated with plasma lipid levels. Alternatively, there might be physiological compensation similar to that observed in PRAP1 knockout mice, which rendered the GWAS study negative for PRAP1.

Last, PRAP1 deficiency or the E85V mutation diminishes hypertriglyceridemia, weight gain, and hepatosteatosis of mice on a HFD. One risk factor for liver cancer is nonalcoholic fatty liver disease, which is characterized in part by excessive accumulation of TG in hepatocytes. This study thus suggests that PRAP1 may serve as a potential therapeutic target for lipid metabolism–related diseases.

## Experimental procedures

### Generation of PRAP1 knockout and E85V knock-in mice

To generate PRAP1 knockout mice, two overlapping genomic fragments harboring the *Prap1* genomic locus were first isolated from a 129/Svj mouse genomic library and used to construct the targeting vector. This targeting vector was constructed by PCR-assisted cloning in such a way that, after homologous recombination, a LacZ reporter linked to a floxed Neo marker would be inserted into exon 3 of the *Prap1* locus. This targeting vector was then electroporated into R1 embryonic stem cells, and Southern blotting using appropriate probes was carried out to select clones that had undergone homologous recombination at the *Prap1* locus. Two positive clones, #159 and #318, harboring the mutant allele were microinjected into C57BL/6 blastocysts to generate chimeric mice. The male chimeric mice were backcrossed with C57BL/6 females to generate PRAP1^*+/*−^ mice. PRAP1^*+/*−^ mice backcrossed to C57BL/6 background for more than 10 generations were intercrossed to generate PRAP1^*+/+*^ (littermate controls) and PRAP1^−/−^ (knockout) mice for this study. Genotyping was performed by PCR with genomic DNA isolated from the mouse tails. PCR primers for the wildtype allele were p1 (5ʹ-TCCAGCACACCAGGTATGCAAGG-3ʹ) and p3 (5ʹ-AGGGTCCTCAAGGGCAAGGGAGT-3ʹ), and the primers for the mutant allele were p1 and p2 (5ʹ-TCGATAGCTTGGCTGCAGGTCG-3ʹ).

To generate the E85V knock-in mutant mice, the CRISPR-Cas–mediated genome engineering system was employed (41). Briefly, the E85V mutation was introduced into the *Prap1* gene through homology-directed repair-mediated genome editing following coinjection of a single-stranded mutant DNA oligo (5ʹ-GTGGGAAGGATCTTGTGAGGGAGGCTATATCTACGTCTCCTTCTCCACAGATGCCATGACATGGGTAGTGACTGAGGATATCCTGAGCCATCTTCGCAGTCCTCTTCAGGGTCCAGAACTGGATCTTGA-3ʹ, mutated sites are underlined), *Prap1*-targeting sgRNA (target site 5ʹ-GATATCCTCAGTCTCCACCC-3ʹ) and Cas9 mRNA into the pronuclear stage one-cell C57BL/6 mouse embryos. By PCR genotyping and genomic sequencing of 23 mice generated, four (#6, #8, #15, and #19) and five (#2, #4, #13, #14, and #18) mice were found to harbor biallelic or monoallelic mutation, respectively. Mice #2 (monoallelic) and #19 (biallelic) were subsequently mated with C57BL/6 mice to generate *PRAP1*^*+/E85V*^ mice. *PRAP1*^*+/E85V*^ mice were then intercrossed to generate *PRAP1*^*+/+*^ (littermate controls) and *PRAP1*^*E85V/E85V*^ (termed as E85V mice) for this study. The E85V mice from both lines (#2 and #19) manifested very similar phenotypes and were analyzed together (∼50% from each line) in statistical analysis. PCR primers for the wildtype allele were PRAP1-I3-F (5ʹ-TACCT ACTCCCTTGCCCTTG-3ʹ) and PRAP1-reverse (5ʹ-CAGTCTCCACCCAGGTCATG-3ʹ), and the primers for the mutant allele were PRAP1-I3-F and E85V-reverse (5ʹ-CAGTCACTACCCATGTCATG-3ʹ).

Mice were housed in a room with a 12-h light/dark cycle (lights on at 7:00 AM) with *ad libitum* access to food and water. We used 8- to 12-week-old male mice for all studies unless otherwise indicated. All animal experiments were performed in accordance with the guidelines set by Academia Sinica Institutional Animal Care and Utilization Committee.

### Mutagenesis, production, and purification of recombinant PRAP1 from bacteria

pGEX4T-1-PRAP1△N20 is a bacterial expression vector that directs the synthesis of PRAP1 without the N-terminal 20-aa signal peptide (PRAP1△N20) C-terminally fused to the glutathione *S*-transferase (GST) protein. Other bacterial expression vectors (also in the pGEX4T-1 vector, Amersham-Pharmacia) expressing various mutants of PRAP1 (*e.g.*, E38V; E51,54V) were constructed by standard PCR-assisted mutagenesis-coupled cloning methods using pGEX4T-1-PRAP1△N20 as a template. p GEX4T-1-PRAP1 21-40, p GEX4T-1-PRAP1 21-76, and p GEX4T-1-PRAP1 21-110 are additional bacterial expression vectors that direct the synthesis of N-terminally GST-tagged PRAP1 mutants covering amino acid residues of PRAP1 as indicated in their names. To purify recombinant PRAP1△N20 (designated as WT rPRAP1 in the text) and various other mutants described above, bacterially produced GST-fusion proteins were purified with glutathione-Sepharose 4B beads (GE Healthcare Life Sciences), digested with thrombin to remove the GST portion, followed by inactivation of thrombin by adding PMSF to a final concentration of 1 mM PMSF. Unless otherwise indicated, all recombinant PRAP1 (WT or mutants, with or without GST tag) mentioned in the text referred to proteins without the N-terminal 20-aa signal peptide.

### Identification of PRAP1-interacting proteins by GST pull-down coupled with mass spectrometry

The proximal one-third of the small intestine of PRAP1^−/−^ mice was opened longitudinally and washed in HBSS (Invitrogen/GIBCO) containing 10 mM DTT for 30 min at RT to remove mucus. The intestinal segments pooled from 10 mice were minced into 1-cm pieces and incubated in HBSS containing 1 mM EDTA (Sigma-Aldrich) for 1 h at 4 °C with vigorous shaking. Isolated IEC were washed twice with PBS and lysed in a buffer (NP-40 lysis buffer) containing 10 mM HEPES, pH 7.5, 142.5 mM KCl, 5 mM MgCl2, 1 mM EGTA, and 0.2% NP-40 supplemented with 1 mM PMSF, 10 μg/ml aprotinin, and 10 μg/ml leupeptin. Bacterially produced GST or GST-PRAP1△N20 (∼10 μg) bound to glutathione beads were added to 2 mg of IEC lysates. After gently mixing for 1 h at 4 °C, beads were washed five times with the NP-40 lysis buffer and proteins retained on the beads were resolved by SDS-PAGE and stained with SYPRO Ruby (Thermo Fisher). Those protein bands appeared in samples pulled-down by GST-PRAP1△N20 but not by GST were processed for mass spectrometric analysis to reveal their identity as previously described (42).

### Generation of PRAP1-specific antibodies

Anti-PRAP1 antibody was generated both in rats and rabbits using bacterially produced recombinant PRAP1△N20. These antibodies were affinity purified by using specific antigen cross-linked to CNBr-activated Sepharose (Amersham).

### Coimmunoprecipitation of MTTP and PRAP1

IEC purified from the small intestine (proximal two-thirds) of control, PRAP1^−/−^, or mutant (E85V) mice were lysed in the homogenization buffer (150 mM NaCl, 1 mM EDTA, 10 mM HEPES, pH 7.4, 0.5 mM PMSF, and 20 μ g/ml leupeptin), and approximately 1 mg lysates were immunoprecipitated with 1 μg of PRAP1 (raised in rat)- or MTTP (Santa Cruz, sc-135994)-specific antibodies. The immunoprecipitated protein complexes were washed three times with the homogenization buffer, resolved by SDS-PAGE, and transferred to the polyvinylidene difluoride membrane (Millipore), and the co-immunoprecipitated proteins were analyzed by immunoblotting with PRAP1 (raised in rabbit)- or MTTP-specific antibodies.

### Detection of MTTP-TG-PRAP1 ternary complex

HeLa cells transiently overexpressing FLAG-tagged MTTP were lysed in the NP-40 lysis buffer, and FLAG-MTTP was immunoprecipitated by FLAG antibody. The immune complex collected on the protein-G agarose was washed three times with the NP-40 lysis buffer, twice with buffer A (50 mM Tris·HCl pH 7.4 and 150 mM NaCl), followed by incubation with recombinant wt or mutant PRAP1 in 500 μl of buffer A containing 0 to 80 pmole of [^3^H]-TG. After incubation for 4 h, the immune complex was washed three times with buffer A and eluted with 200 μl of 0.1 M glycine (pH 2.5). The eluate was immediately neutralized with 40 μl of 1 M Tris. HCl (pH 9.0). Fifty microliters of the neutralized sample was subjected to isotope counting for ^3^H-TG, and the rest (∼190 μl) was processed to remove lipids by precipitation of proteins with cold acetone according to a published protocol (43). The precipitated proteins were resuspended in a buffer containing 20 mM Tris, 1 mM EDTA, pH7.4, and 1% SDS, followed by SDS-PAGE and immunoblotting analysis of FLAG-tagged MTTP and recombinant GST or GST-PRAP1 (wt or mutant) using anti-FLAG or anti-GST antibodies, respectively.

### *In vitro* MTTP activity assays

MTTP activities were measured using the Roar MTTP activity Assay kit (Roar Biomedical, New York, NY, USA) according to the manufacturer's instructions. Briefly, IECs from control or PRAP1^−/−^ mice resuspended in homogenization buffer (150 mM NaCl, 1 mM EDTA, 10 mM Tris, pH 7.4, 0.5 mM PMSF, and 20 μg/ml leupeptin) were sonicated on ice (five 5-s bursts in a 550-W sonicator; power setting: 4). Two hundred micrograms of homogenates was then incubated with 4 μl of donor particle and 4 μl of acceptor particle in 0.2 ml total volume with reconstituted assay buffer. Sixteen hours after the incubation step, the fluorescence intensity (reflecting the amount of triglyceride or phospholipids being transferred) in the sample was determined by fluorimeter (Ex: 465 nm; Em: 535 nm). A blank not containing cell homogenates was included as a background for this type of assay.

### Measurements of plasma levels of triglyceride, phospholipids, cholesterol, or free fatty acids

Plasma samples were collected from mice to be measured by standard methods, and the amounts of TGs, phospholipids, TCHO, or free fatty acids were determined enzymatically using commercial kits from Fujifilm (Tokyo, Japan) or Biovision (Milpitas, CA, USA).

### Measurement of lipid absorption in the small intestines

Mice to be tested were provided with food only during 7 PM to 7 AM for 3 days. On the fourth day at 7 PM, mice were i.v. injected with 500 mg/kg body weight Tyloxapol (to block hydrolysis of TG-rich lipoproteins), followed by an intragastric bolus of 0.5 ml of corn oil containing 5 μCi of [^14^C]-Triolein and 1 μCi of [^3^H]-β-sitostanol (serves as a control in that it can hardly be absorbed by the small intestine) (44). One to 3 h later, the middle portion of the small intestine was collected, the radioactive contents in the cell lysates were measured, and the ratio of [^14^C]/[^3^H] radioactivity was determined and plotted. In some cases, blood samples were collected at 1 to 3 h after lipid administration, and the amount of [^14^C]-Triolein–derived radioactivity in the whole blood was determined. For counting the radioactivity in either the small intestine or the blood sample, the tissue specimens were solubilized first by the Soluene-350 method as detailed in the manufacturer's protocol prior to the addition of the scintillation cocktail Ultima Gold (PerkinElmer, USA).

### Lipid binding assays

Binding of [^3^H]-TG (triolein, [9,10-^3^H(N)], PerkinElmer) to recombinant WT or mutant PRAP1 proteins was carried out essentially as described (45) with minor modifications. Briefly, approximately 0.7 μg of GST or GST-PRAP1 proteins (WT or mutant, all without signal peptides unless otherwise indicated) bound to glutathione-Sepharose 4B beads (Pharmacia) was washed with buffer A (50 mM Tris·HCl pH 7.4 and 150 mM NaCl) three times and then resuspended in 100 μl of buffer A containing the indicated amounts of [^3^H]-TG. After 4 h incubation at room temperature (RT), beads were washed three times with 800 μl of buffer A by centrifugation and resuspended in 500 μl of buffer E (10 mM Tris·HCl pH 7.4, 150 mM NaCl, 1 mM EDTA, and 1% Triton X-100), and assayed for retained radioactivity using a scintillation counter.

### Pulse chase and radiolabeling experiments

This experiment was carried out essentially as described (46) with minor modifications. Briefly, IECs (10^7^/reaction) isolated from the indicated genotypes were pulse labeled for 30 min with 250 μCi/ml EXPRE^35^S^35^S Protein Labeling Mix (PerkinElmer) and chased for 0 to 120 min in Dulbecco's modified Eagle's medium supplemented with 10 mM methionine and 5 mM cysteine. A mixed micellar lipid solution (final concentrations [in mM]: 0.4 sodium taurocholate, 0.54 sodium taurodeoxycholate, 0.3 phosphatidylcholine, 0.45 oleic acid, 0.26 monoolein) was added to both pulse and chase media. Following the pulse–chase steps, both cell lysates and medium were immunoprecipitated with apoB antibody and the immune complexes resolved by 5% SDS-PAGE and fluorography. The signals were revealed by the use of Typhoon FLA 9000 biomolecular imager (GE Healthcare).

For metabolic labeling of mouse primary hepatocytes, we essentially followed the protocol as described (27) with minor modifications. Briefly, 3 h after hepatocytes were plated (6 × 10^5^ per 6-well collagen-coated tissue culture plate), 50 μl of 250 μCi/ml EXPRE^35^S^35^S Protein Labeling Mix (PerkinElmer) was added to each well. After 3 h labeling at 37 °C (prior tests showed that this is within linear range), both cell lysates and medium were immunoprecipitated with apoB antibody and the immune complexes resolved by 5% SDS-PAGE and fluorography. The signals were revealed by the use of Typhoon FLA 9000 biomolecular imager (GE Healthcare).

### Real-time quantitative PCR

Total RNA from samples of interest was isolated with the TRIzol reagent (Thermo Fisher). Isolated RNA was then reverse transcribed into cDNA using random hexamer and SuperScript III reverse transcriptase (Invitrogen). The mRNA expression levels of genes of interest were analyzed by real-time quantitative PCR (RT-qPCR) on a LightCycler Real-Time PCR system according to the manufacturer's protocol (Roche Applied Science, Indianapolis, IN, USA). Sequences of the primers used in this assay are shown in Table S1.

### Density gradient ultracentrifugation

HeLa cells (6 × 10^5^ cells in 6-cm plate) were cotransfected with ApoB48, MTTP along with PRAP1-WT or E85V-expressing vector by Lipofectamine 3000 (Invitrogen) following manufacture's instruction. After 24 h, the medium was aspirated and replaced with 4.5 ml lipid-rich medium (Dulbecco's modified Eagle's medium/1% fatty acid–free bovine serum albumin/0.8 mM sodium oleate/1 mM glycerol/0.05 mg/ml of cholesterol) for another 24 h. After harvesting, media were adjusted to contain 1 mM PMSF, 0.02%EDTA, 0.02% NaN3, and Protease inhibitor cocktail (Roche), and centrifuged for 5 min at 1200 rpm to remove cell debris. ApoB-containing lipoproteins in the conditioned medium were separated by density gradient ultracentrifugation as described (47) with minor modifications. Briefly, 4 ml of the medium, adjusted to d = 1.12 g/ml with solid KBr (0.565 g), was sequentially overlaid with 3 ml of d = 1.063 g/ml, 3 ml of d = 1.019 g/ml, and 2 ml of d = 1.006 g/ml density solutions in a Beckman SW41 centrifuge tube. After centrifugation at 40,000 rpm for 33 min at 15 °C, the top 1 ml of the gradient was collected as fraction 1. The gradients were then overlaid with 1 ml of d = 1.006 g/ml density solutions. Following subsequent centrifugation at 40,000 rpm for 3 h 30 min at 15 °C, the top 1 ml of the gradient was collected as fraction 2. The tubes were overlaid with 1 ml of d = 1.006 g/ml density solutions and centrifuged at 40,000 rpm for 17 h at 15 °C. Other lipoproteins were collected from the top into twelve 1-ml fractions. Proteins in individual fractions were processed to remove lipids by precipitation of proteins with cold acetone according to a published protocol (43). The precipitated proteins were resuspended in a buffer containing 20 mM Tris, 1 mM EDTA, pH7.4, and 1% SDS, followed by SDS-PAGE and immunoblotting analysis using MTTP-, apoB-, and PRAP1-specific antibodies.

### Fractionation of plasma lipoproteins

Pooled plasma from three mice of the indicated genotypes (300 μl) (actual injection volume 200 μl) was fractionated by fast phase liquid chromatography using a single Superose 6HR 10/30 column as described (23). Elution was performed in phosphate-buffered saline containing 0.01% EDTA and 0.02% NaN3, and approximately 0.6 ml was collected for each fraction. Fractions 12 to 31 were collected for lipid and protein analyses. In some cases, pooled plasma (0.6–1 ml) from 3 to 5 mice of the indicted genotypes were fractionated by density gradient ultracentrifugation essentially as described (21). Briefly, 1 ml of plasma was adjusted to d = 1.10 g/ml with solid NaCl (140.4 mg) and mixed with 3 ml of 1.1 g/ml NaCl in a Beckman SW41 centrifuge tube. A density gradient consisting of 3 ml of d = 1.065 g/ml NaCl, 3 ml of d = 1.02 g/ml NaCl, and 2 ml of d = 1.006 g/ml NaCl was then sequentially layered on top of the plasma. After centrifugation at 40,000 rpm for 33 min at 15 °C, the top 1 ml of the gradient was collected as fraction 1 (large chylomicrons, d = ∼0.96 g/ml). The gradient was then overlaid with 1 ml of 1.006 g/ml solution and centrifuged again at 40,000 rpm for 3 h and 30 min (15 °C). The top 1 ml was collected as fraction 2 (small chylomicrons). The gradient was replenished with 1.006 g/ml solutions and centrifuged again (40,000 rpm, 15 °C, 17 h). The top 1 ml was collected as fraction 3 (VLDL size particles). The rest of the gradient was collected into ten 1-ml fractions.

### Bomb calorimetry

Feces collected from individually housed mice over 48 h were dried at 60 °C for 2 days, homogenized, and subjected to analysis in triplicates (0.2 μg, each) using Parr 6725 semimicro oxygen bomb calorimeter. Energy content per gram of dry weight was then calculated.

### Determination of fecal lipid content

Feces over 2 to 4 days were collected from individually housed mice that had been fed a HFD for 2 weeks according to a published protocol (48).

### Statistics

Statistical analysis was performed with either *t* tests for comparison between two groups or one-way analysis of variance (Tukey's post test) for comparison among multiple experimental groups using GraphPad Prism 8 software (San Diego).

## Data availability

All data are contained within the article and associated Supporting Information.

## Conflict of interest

The authors declare that they have no conflicts of interest with the contents of this article.

## References

[1] Hooper A.J., Burnett J.R., Watts G.F. (2015). Contemporary aspects of the biology and therapeutic regulation of the microsomal triglyceride transfer protein. Circ. Res..

[2] Greeve J., Altkemper I., Dieterich J.H., Greten H., Windler E. (1993). Apolipoprotein B mRNA editing in 12 different mammalian species: hepatic expression is reflected in low concentrations of apoB-containing plasma lipoproteins. J. Lipid Res..

[3] Wetterau J.R., Lin M.C., Jamil H. (1997). Microsomal triglyceride transfer protein. Biochim. Biophys. Acta.

[4] Wetterau J.R., Zilversmit D.B. (1986). Localization of intracellular triacylglycerol and cholesteryl ester transfer activity in rat tissues. Biochim. Biophys. Acta.

[5] Iqbal J., Walsh M.T., Hammad S.M., Cuchel M., Tarugi P., Hegele R.A., Davidson N.O., Rader D.J., Klein R.L., Hussain M.M. (2015). Microsomal triglyceride transfer protein transfers and determines plasma concentrations of ceramide and sphingomyelin but not glycosylceramide. J. Biol. Chem..

[6] Rava P., Hussain M.M. (2007). Acquisition of triacylglycerol transfer activity by microsomal triglyceride transfer protein during evolution. Biochemistry.

[7] Wetterau J.R., Zilversmit D.B. (1984). A triglyceride and cholesteryl ester transfer protein associated with liver microsomes. J. Biol. Chem..

[8] Hussain M.M., Rava P., Walsh M., Rana M., Iqbal J. (2012). Multiple functions of microsomal triglyceride transfer protein. Nutr. Metab. (Lond.).

[9] Sirwi A., Hussain M.M. (2018). Lipid transfer proteins in the assembly of apoB-containing lipoproteins. J. Lipid Res..

[10] Lamberg A., Jauhiainen M., Metso J., Ehnholm C., Shoulders C., Scott J., Pihlajaniemi T., Kivirikko K.I. (1996). The role of protein disulphide isomerase in the microsomal triacylglycerol transfer protein does not reside in its isomerase activity. Biochem. J..

[11] Hooper A.J., van Bockxmeer F.M., Burnett J.R. (2005). Monogenic hypocholesterolaemic lipid disorders and apolipoprotein B metabolism. Crit. Rev. Clin. Lab. Sci..

[12] Zamel R., Khan R., Pollex R.L., Hegele R.A. (2008). Abetalipoproteinemia: two case reports and literature review. Orphanet J. Rare Dis..

[13] Xie Y., Newberry E.P., Young S.G., Robine S., Hamilton R.L., Wong J.S., Luo J., Kennedy S., Davidson N.O. (2006). Compensatory increase in hepatic lipogenesis in mice with conditional intestine-specific Mttp deficiency. J. Biol. Chem..

[14] Biterova E.I., Isupov M.N., Keegan R.M., Lebedev A.A., Sohail A.A., Liaqat I., Alanen H.I., Ruddock L.W. (2019). The crystal structure of human microsomal triglyceride transfer protein. Proc. Natl. Acad. Sci. U. S. A..

[15] Kasik J., Rice E. (1997). A novel complementary deoxyribonucleic acid is abundantly and specifically expressed in the uterus during pregnancy. Am. J. Obstet. Gynecol..

[16] Lepourcelet M., Tou L., Cai L., Sawada J., Lazar A.J., Glickman J.N., Williamson J.A., Everett A.D., Redston M., Fox E.A., Nakatani Y., Shivdasani R.A. (2005). Insights into developmental mechanisms and cancers in the mammalian intestine derived from serial analysis of gene expression and study of the hepatoma-derived growth factor (HDGF). Development.

[17] Zhang J., Rajkumar N., Hooi S.C. (2000). Characterization and expression of the mouse pregnant specific uterus protein gene and its rat homologue in the intestine and uterus. Biochim. Biophys. Acta.

[18] Zhang J., Wong H., Ramanan S., Cheong D., Leong A., Hooi S.C. (2003). The proline-rich acidic protein is epigenetically regulated and inhibits growth of cancer cell lines. Cancer Res..

[19] Huang B.H., Zhuo J.L., Leung C.H., Lu G.D., Liu J.J., Yap C.T., Hooi S.C. (2012). PRAP1 is a novel executor of p53-dependent mechanisms in cell survival after DNA damage. Cell Death Dis..

[20] Gordon D.A., Jamil H., Sharp D., Mullaney D., Yao Z., Gregg R.E., Wetterau J. (1994). Secretion of apolipoprotein B-containing lipoproteins from HeLa cells is dependent on expression of the microsomal triglyceride transfer protein and is regulated by lipid availability. Proc. Natl. Acad. Sci. U. S. A..

[21] Karpe F., Hamsten A., Uffelman K., Steiner G. (1996). Apolipoprotein B-48. Methods Enzymol..

[22] Wetterau J.R., Aggerbeck L.P., Bouma M.E., Eisenberg C., Munck A., Hermier M., Schmitz J., Gay G., Rader D.J., Gregg R.E. (1992). Absence of microsomal triglyceride transfer protein in individuals with abetalipoproteinemia. Science.

[23] Innis-Whitehouse W., Li X., Brown W.V., Le N.A. (1998). An efficient chromatographic system for lipoprotein fractionation using whole plasma. J. Lipid Res..

[24] Atzel A., Wetterau J.R. (1993). Mechanism of microsomal triglyceride transfer protein catalyzed lipid transport. Biochemistry.

[25] Atzel A., Wetterau J.R. (1994). Identification of two classes of lipid molecule binding sites on the microsomal triglyceride transfer protein. Biochemistry.

[26] Raabe M., Flynn L.M., Zlot C.H., Wong J.S., Véniant M.M., Hamilton R.L., Young S.G. (1998). Knockout of the abetalipoproteinemia gene in mice: reduced lipoprotein secretion in heterozygotes and embryonic lethality in homozygotes. Proc. Natl. Acad. Sci. U. S. A..

[27] Raabe M., Veniant M.M., Sullivan M.A., Zlot C.H., Bjorkegren J., Nielsen L.B., Wong J.S., Hamilton R.L., Young S.G. (1999). Analysis of the role of microsomal triglyceride transfer protein in the liver of tissue-specific knockout mice. J. Clin. Invest..

[28] Abumrad N.A., Davidson N.O. (2012). Role of the gut in lipid homeostasis. Physiol. Rev..

[29] Cartwright I.J., Higgins J.A. (2001). Direct evidence for a two-step assembly of ApoB48-containing lipoproteins in the lumen of the smooth endoplasmic reticulum of rabbit enterocytes. J. Biol. Chem..

[30] Rustaeus S., Lindberg K., Stillemark P., Claesson C., Asp L., Larsson T., Boren J., Olofsson S.O. (1999). Assembly of very low density lipoprotein: a two-step process of apolipoprotein B core lipidation. J. Nutr..

[31] Kulinski A., Rustaeus S., Vance J.E. (2002). Microsomal triacylglycerol transfer protein is required for lumenal accretion of triacylglycerol not associated with ApoB, as well as for ApoB lipidation. J. Biol. Chem..

[32] Wang Y., Tran K., Yao Z. (1999). The activity of microsomal triglyceride transfer protein is essential for accumulation of triglyceride within microsomes in McA-RH7777 cells. A unified model for the assembly of very low density lipoproteins. J. Biol. Chem..

[33] Teslovich T.M., Musunuru K., Smith A.V., Edmondson A.C., Stylianou I.M., Koseki M., Pirruccello J.P., Ripatti S., Chasman D.I., Willer C.J., Johansen C.T., Fouchier S.W., Isaacs A., Peloso G.M., Barbalic M. (2010). Biological, clinical and population relevance of 95 loci for blood lipids. Nature.

[34] Willer C.J., Schmidt E.M., Sengupta S., Peloso G.M., Gustafsson S., Kanoni S., Ganna A., Chen J., Buchkovich M.L., Mora S., Beckmann J.S., Bragg-Gresham J.L., Chang H.Y., Demirkan A., Den Hertog H.M. (2013). Discovery and refinement of loci associated with lipid levels. Nat. Genet..

[35] Beck T., Hastings R.K., Gollapudi S., Free R.C., Brookes A.J. (2014). GWAS Central: a comprehensive resource for the comparison and interrogation of genome-wide association studies. Eur. J. Hum. Genet..

[36] Welter D., MacArthur J., Morales J., Burdett T., Hall P., Junkins H., Klemm A., Flicek P., Manolio T., Hindorff L., Parkinson H. (2014). The NHGRI GWAS Catalog, a curated resource of SNP-trait associations. Nucleic Acids Res..

[37] Ramos E.M., Hoffman D., Junkins H.A., Maglott D., Phan L., Sherry S.T., Feolo M., Hindorff L.A. (2014). Phenotype-Genotype Integrator (PheGenI): synthesizing genome-wide association study (GWAS) data with existing genomic resources. Eur. J. Hum. Genet..

[38] Barrett T., Suzek T.O., Troup D.B., Wilhite S.E., Ngau W.C., Ledoux P., Rudnev D., Lash A.E., Fujibuchi W., Edgar R. (2005). NCBI GEO: mining millions of expression profiles--database and tools. Nucleic Acids Res..

[39] Schorderet D.F. (1991). Using OMIM (On-line Mendelian Inheritance in Man) as an expert system in medical genetics. Am. J. Med. Genet..

[40] Koscielny G., An P., Carvalho-Silva D., Cham J.A., Fumis L., Gasparyan R., Hasan S., Karamanis N., Maguire M., Papa E., Pierleoni A., Pignatelli M., Platt T., Rowland F., Wankar P. (2017). Open Targets: a platform for therapeutic target identification and validation. Nucleic Acids Res..

[41] Wang H., Yang H., Shivalila C.S., Dawlaty M.M., Cheng A.W., Zhang F., Jaenisch R. (2013). One-step generation of mice carrying mutations in multiple genes by CRISPR/Cas-mediated genome engineering. Cell.

[42] Huang C.R., Yang-Yen H.F. (2010). The fast-mobility isoform of mouse Mcl-1 is a mitochondrial matrix-localized protein with attenuated anti-apoptotic activity. FEBS Lett..

[43] Kozlitina J., Smagris E., Stender S., Nordestgaard B.G., Zhou H.H., Tybjaerg-Hansen A., Vogt T.F., Hobbs H.H., Cohen J.C. (2014). Exome-wide association study identifies a TM6SF2 variant that confers susceptibility to nonalcoholic fatty liver disease. Nat. Genet..

[44] Mera Y., Odani N., Kawai T., Hata T., Suzuki M., Hagiwara A., Katsushima T., Kakutani M. (2011). Pharmacological characterization of diethyl-2-({3-dimethylcarbamoyl-4-[(4'-trifluoromethylbiphenyl-2-carbonyl)amino]p henyl}acetyloxymethyl)-2-phenylmalonate (JTT-130), an intestine-specific inhibitor of microsomal triglyceride transfer protein. J. Pharmacol. Exp. Ther..

[45] Gross D.A., Zhan C., Silver D.L. (2011). Direct binding of triglyceride to fat storage-inducing transmembrane proteins 1 and 2 is important for lipid droplet formation. Proc. Natl. Acad. Sci. U. S. A..

[46] Xie Y., Nassir F., Luo J., Buhman K., Davidson N.O. (2003). Intestinal lipoprotein assembly in apobec-1-/- mice reveals subtle alterations in triglyceride secretion coupled with a shift to larger lipoproteins. Am. J. Physiol. Gastrointest. Liver Physiol..

[47] Iqbal J., Anwar K., Hussain M.M. (2003). Multiple, independently regulated pathways of cholesterol transport across the intestinal epithelial cells. J. Biol. Chem..

[48] Kraus D., Yang Q., Kahn B.B. (2015). Lipid extraction from mouse feces. Bio. Protoc..

