# Supplementary material for: PRAP1 is a novel lipid-binding protein that promotes lipid absorption by facilitating MTTP-mediated lipid transport
Source: J Biol Chem. 2020 Nov 24;296:100052. doi: 10.1074/jbc.RA120.015002 (PMC7949078; doi:10.1074/jbc.RA120.015002)
Supplement: Supplementary file 1 — Figures S1–S9 and Tables S1 and S2 [file mmc1.pdf]

Supporting Information

PRAP1 is a novel lipid binding protein that promotes lipid absorption by facilitating MTTP-mediated lipid transport

Hubert Peng[1], Tzu-Yuan Chiu[1], Yu-Jen Liang[2], Chia-Jen Lee[1], Chih-Syuan Liu[1], Ching-Shu Suen[3], Jeffrey J.-Y. Yen[3], Hung-Ta Chen[1], Ming-Jing Hwang[3], M. Mahmood Hussain[4], Hsin-Chou Yang[2] and Hsin-Fang Yang-Yen[1]

[1] Institute of Molecular Biology, [2]Institute of Statistical Science and [3]Institute of Biomedical Sciences, Academia Sinica, Taipei, Taiwan; [4] Foundations of Medicine, NYU Long Island School of Medicine, Mineola, New York, USA.

This file contains Supplementary Figures S1-S9, and Tables S1-S2.

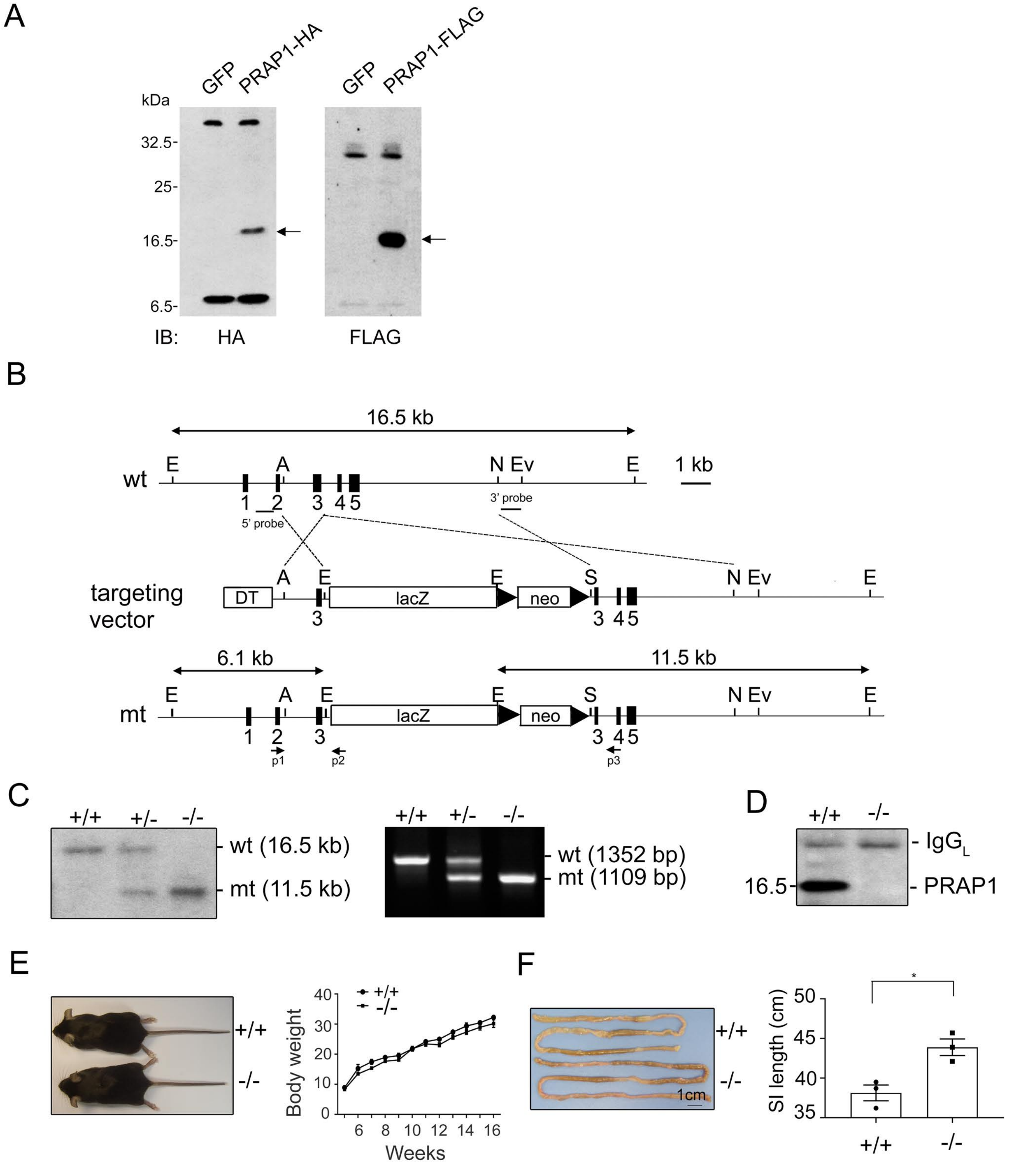

Figure S1. PRAP1 is a secreted protein and PRAP1-deficent mice have a longer small intestine. (A) Conditioned medium from CHOP cells transiently transfected with HA or FLAG-tagged PRAP1 (PRAP1-HA or PRAP1-FLAG, both tags are at the C-terminus) expression vectors was analyzed by immunoblot using HA or FLAG-tag antibody as indicated. Conditioned medium from cells transfected with a control vector (GFP) was included as a negative control. Arrows point to tagged PRAP1. (B) Genomic structure and restriction map of the wild-type mouse Prap1 gene locus. Exons are numbered and represented by solid boxes. Shown below the genomic map are the structures of the targeting vector, and the mutant allele after homologous recombination. The DT gene and the lacZ-neo cassette for negative and positive selection, respectively, and some relevant restriction sites are as indicated (A, Apa I; E, EcoRI; S, Sal I; Ev, EcoR V; N, NdeI). The 5' and 3' probes and the predicted length of EcoRI restriction fragments in Southern blot analysis are as indicated. (C) (Left panel) Southern blot analysis of the tail DNA from wild-type (+/+), heterozygous (+/-) and knockout (-/-) mice. Genomic DNA extracted from mice with the indicated genotype was digested with EcoR I and probed with the 3' probe. The predicted signals for the wild-type (wt) and targeted allele (mt) are as indicated. (Right panel) Genotyping of the indicated mice were carried out by PCR using primers p1 and p3 for the wt allele (1352 bp), and p1 and p2 for the mutant allele (1109 bp). (D) Immunoblotting analysis of small intestinal extracts from representative +/+ and -/- mice. The small intestinal extracts from mice with the indicated genotype were immunoprecipitated with anti-PRAP1 antibody. The precipitated immune complexes were then analyzed by immunoblotting using the same antibody. The signals for PRAP1 and the light chain of the immunoglobulin are as indicated. (E) (Left panel) Representative gross morphology of control and PRAP1-deficint mice. (Right panel) Body weight of control or PRAP1$^{-/-}$ mice on a chow diet for 12 weeks was measured and plotted against time. N=10 for each genotype. (F) Representative small intestines of control and PRAP1$^{-/-}$ mice. *, P<0.05.

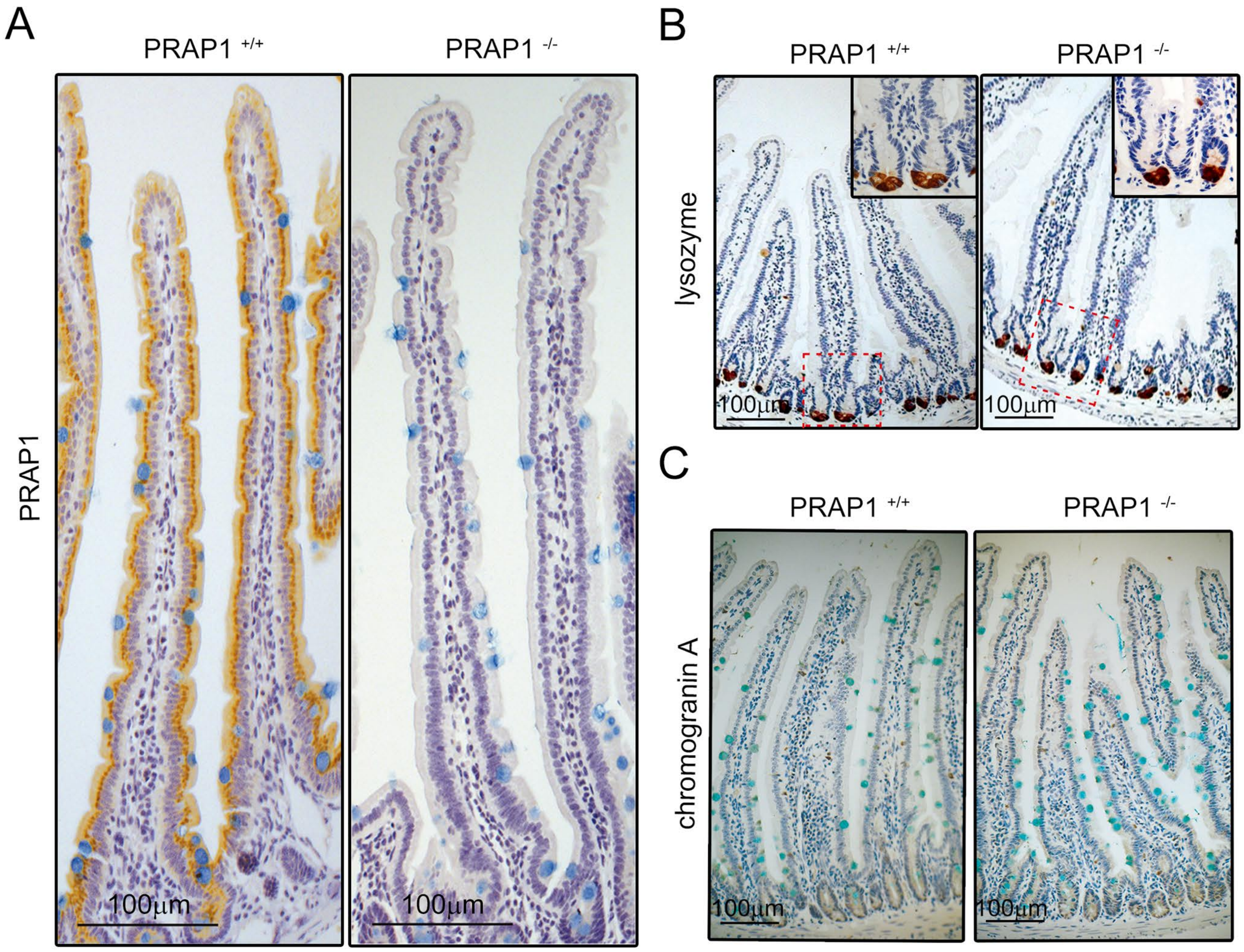

Figure S2. Normal differentiation of intestinal epithelium of PRAP1 deficient mice. (A) Immunohistochemical (IHC) analysis of PRAP1 expression (stained brown) in the small intestines of PRAP1$^{+/+}$ and PRAP1$^{-/-}$ mice using anti-PRAP1 antibody. Sections were counterstained with hematoxylin and alcian blue to reveal nuclei and goblet cells, respectively. (B,C) IHC analysis of small intestinal sections from PRAP1$^{+/+}$ and PRAP1$^{-/-}$ mice using antibodies specific to markers for the Paneth cell (lysozyme, panel B) or enteroendocrine cells (chromogranin A, panel C).

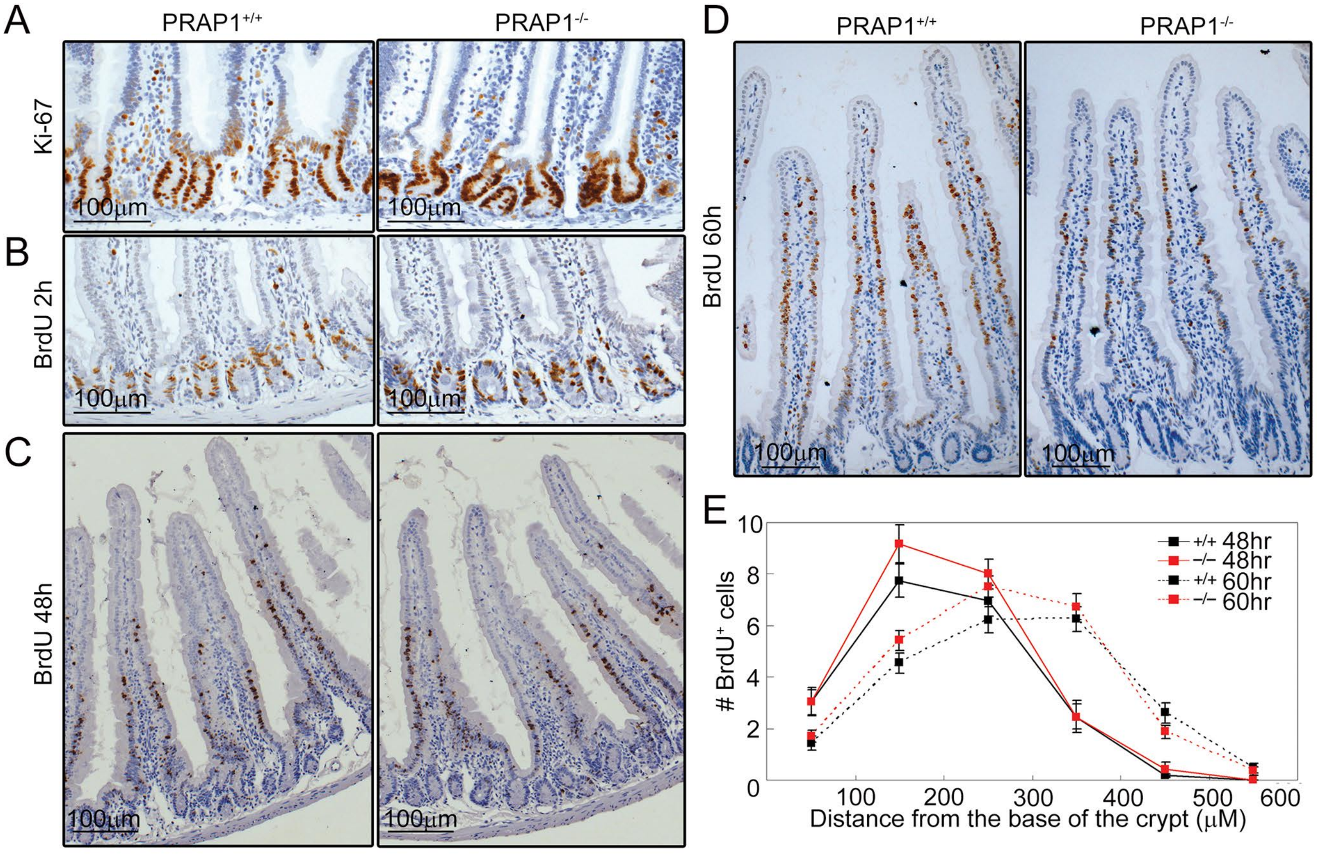

Figure S3. Deficiency of PRAP1 did not significantly affect proliferation or migration of epithelial cells in the small intestine. Immunohistochemical analysis of small intestinal sections from mice with the indicated genotype using antibodies specific for the proliferation marker Ki-67 (A) or BrdU (B-D). For panels B-D, tissue sections were prepared from mice pulsed with BrdU for 2 hrs (B) or sacrificed 48 (C) or 60 (D) hrs later following the BrdU injection. (E) Quantification of BrdU-positive cell numbers in the specified region of the small intestinal villi (every 100-um from the base of the crypt). Thirty to 70 villi from 3-7 mice of each genotype at 48- or 60-hr time point were counted.

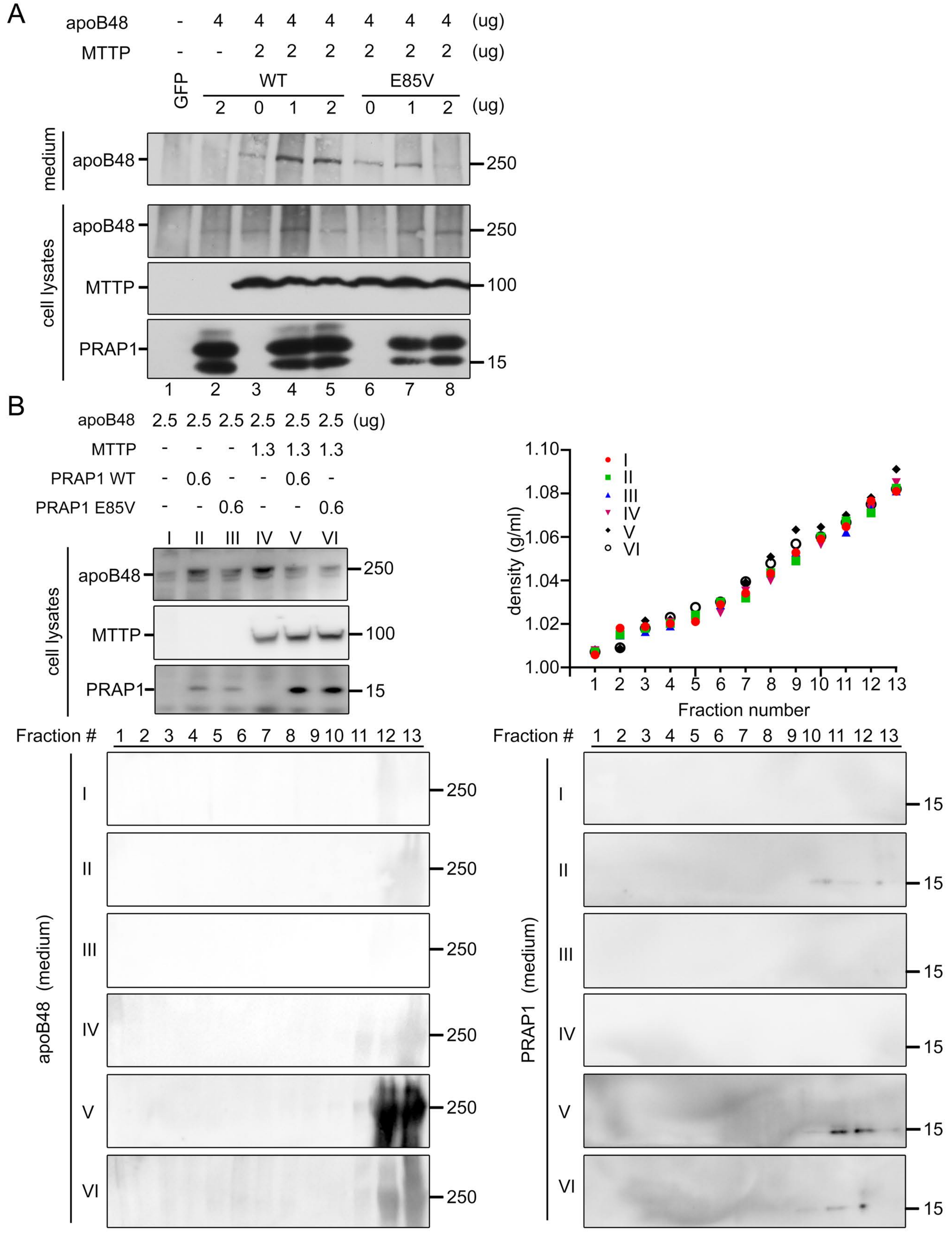

Figure S4. PRAP1 facilitates MTTP-mediated apoB48 secretion using the transient transfection system. (A) HeLa cells were transiently transfected with expression vectors encoding MTTP and apoB48 along with increasing amounts of PRAP1 (WT or the E85V mutant) expression vectors. ApoB48 secreted into the culture medium was analyzed by IP/western using apoB specific antibody. (B) The experiment was carried out essentially as that described in (A), except that culture medium was fractionated by density gradient ultracentrifugation and the proteins in each fraction were analyzed by immunoblotting using apoB or PRAP1 specific antibodies. (I-VI) denote the cells transfected with the indicated amounts (ug) of apoB, MTTP or PRAP1 (WT or E85V) expression vectors. Gradient density is shown in the upper right panel.

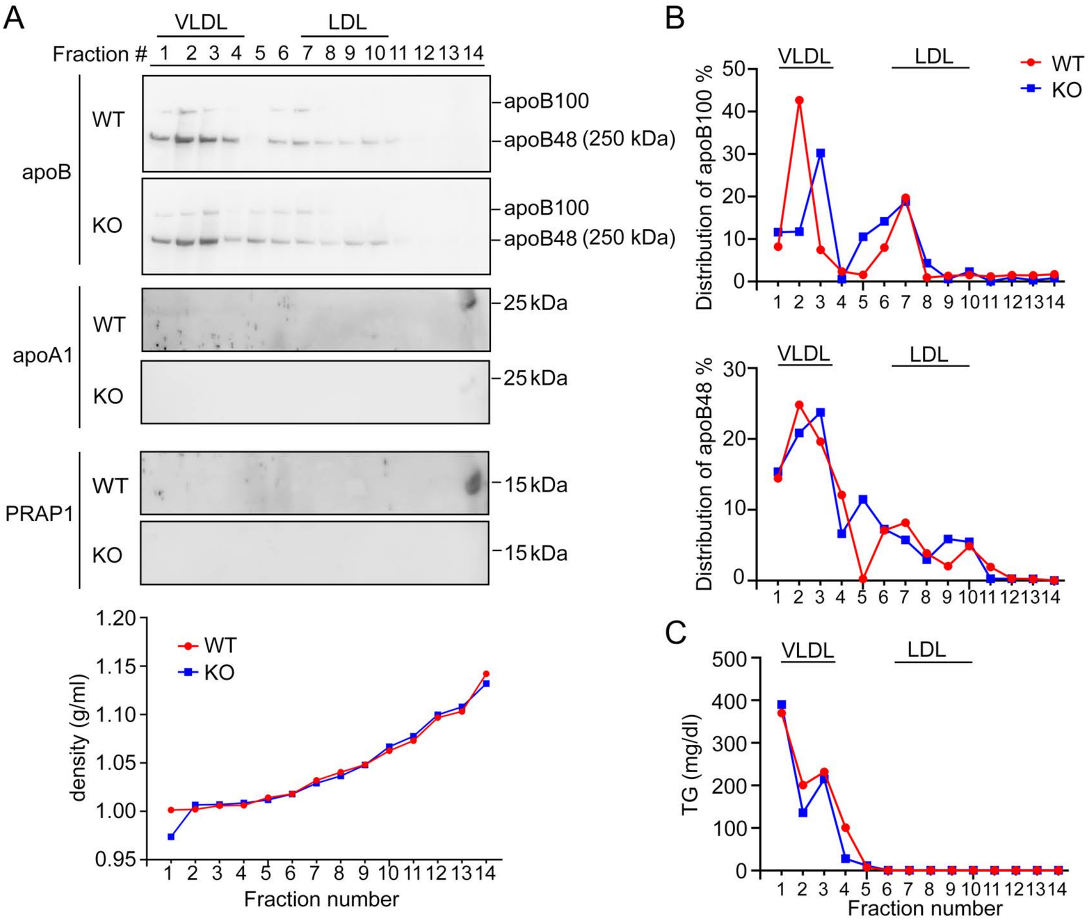

Figure S5. PRAP1 deficiency slightly reduces apoB lipidation and secretion into hepatic VLDL. Control (WT) or PRAP1$^{-/-}$ (KO) mice were fasted for 16 hrs before tyloxapol injection. Three hours later, the plasma (800 ul) pooled from four mice of the same genotype was fractionated by density gradient ultracentrifugation, followed by immunoblotting analysis of the indicated protein (A) or measurement of TG levels (C) in each fraction. The apoB protein signal in each fraction was quantitated and plotted as a relative percentage of total apoB from all 20 fractions from WT plasma (B). The density of each fraction is shown at the bottom of panel A.

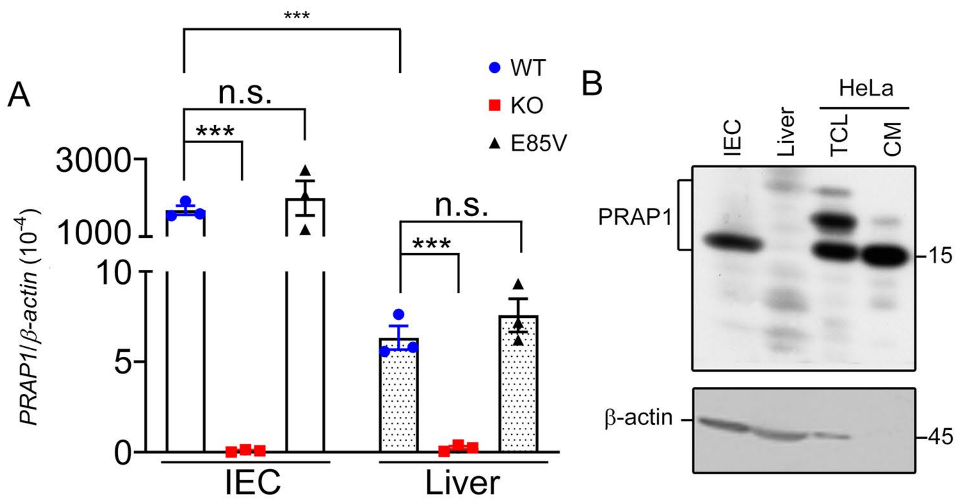

Figure S6. PRAP1 is predominantly expressed in IEC than in livers. (A) Quantitative RT-PCR analysis of the mRNA levels of PRAP1 in purified IEC and livers from mice with the indicated genotype. (B) Immunoblotting analysis of PRAP1 protein expression from IEC, liver, cell lysates (TCL) or conditioned medium (CM) from HeLa cells transiently overexpressing PRAP1.

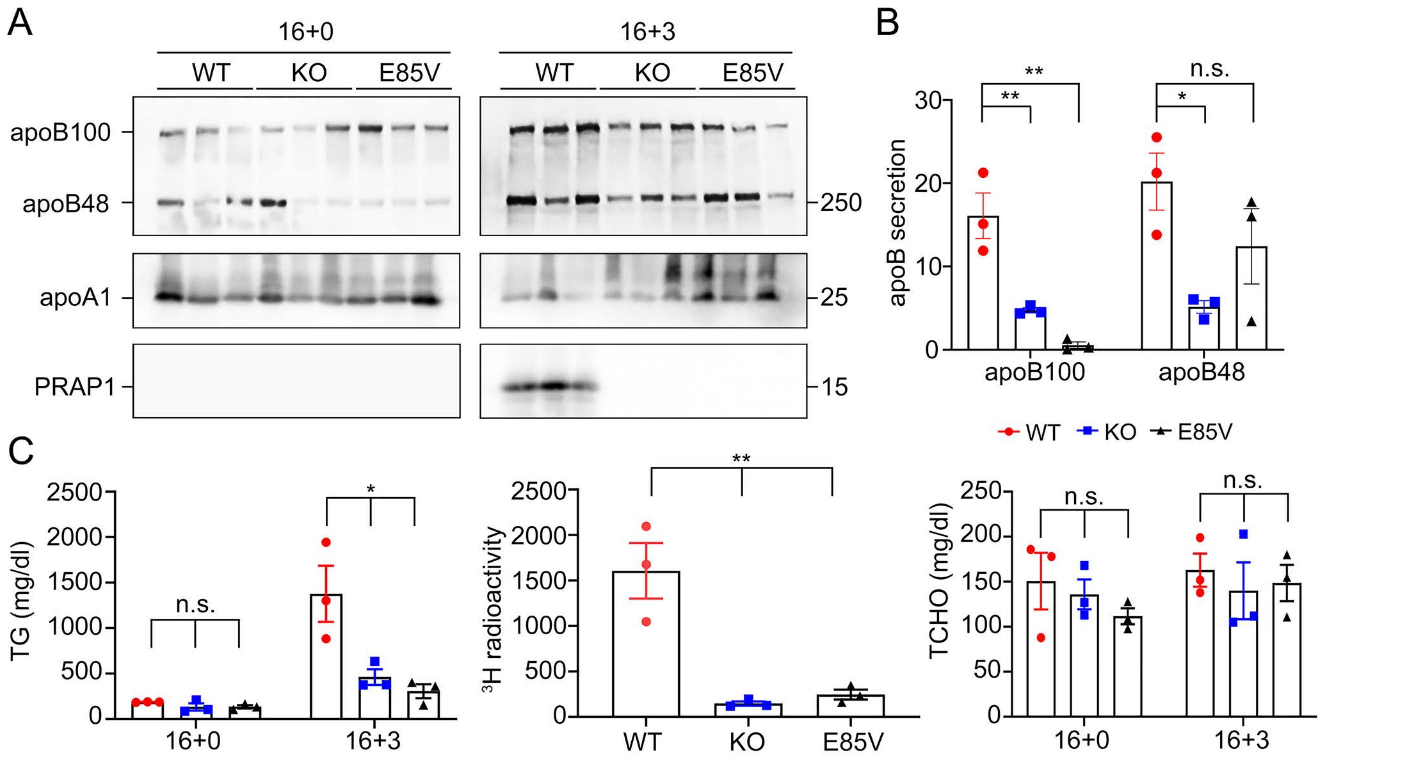

Figure S7. The E85V mutation reduces apoB-lipoprotein assembly and secretion. Plasma were collected from mice with the indicated genotypes after a 16h fast (16+0) or from the same mice receiving additional oral gavage of lipid emulsion containing 10 uCi of [^{3}H] triolein for 3 hrs immediately following tyloxapol injection (16+3). (A) Plasma levels of apoB from "16+0" or "16+3" treated mice of the indicated genotypes (3 for each). (B) The amount of apoB secretion during those 3h post-lipid administration was calculated by subtracting the protein levels detected in the same mouse right after the 16h-fasting. (C) TG mass, [^{3}H] radioactivity and TCHO levels from each individual mouse plasma analyzed in (A).

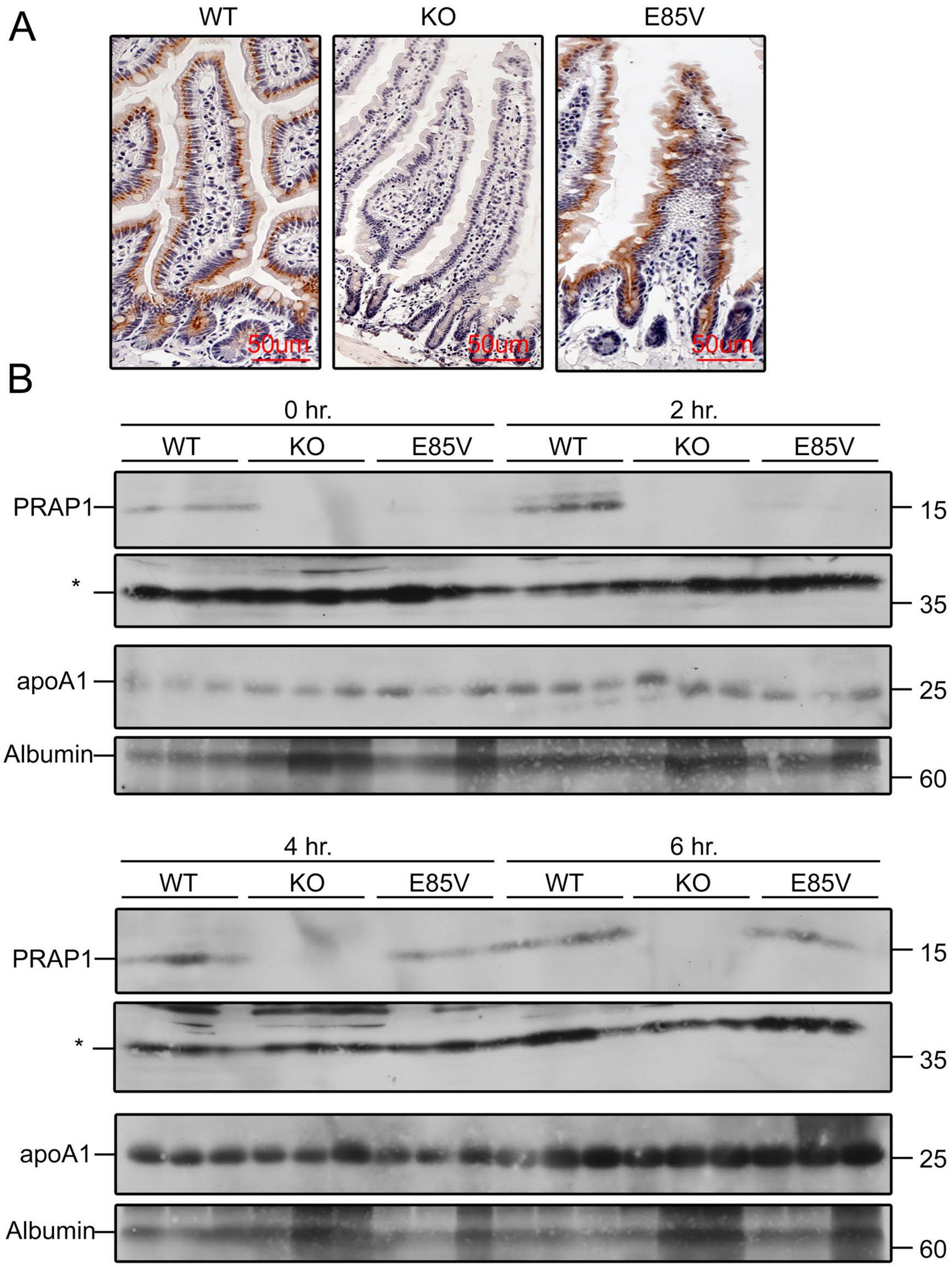

Figure S8. (A) The E85V mutant protein is expressed in intestinal epithelial cells. The small intestinal sections from mice with the indicated genotypes were analyzed by immunohistochemical analysis using PRAP1 specific antibodies. (B) Plasma levels of PRAP1 (WT, KO or the E85V mutant) at various time points following lipid administration were analyzed by immunoblotting analysis. Mice (three for each genotype) were fasted for 4 hrs before oral gavage of lipids. The same plasma was run as two blots, one was probed with PRAP1 antibody (upper), and the other was probed with apoA1 and albumin antibodies. *, an unknown protein cross-reacted with the PRAP1 antibody served as a loading control.

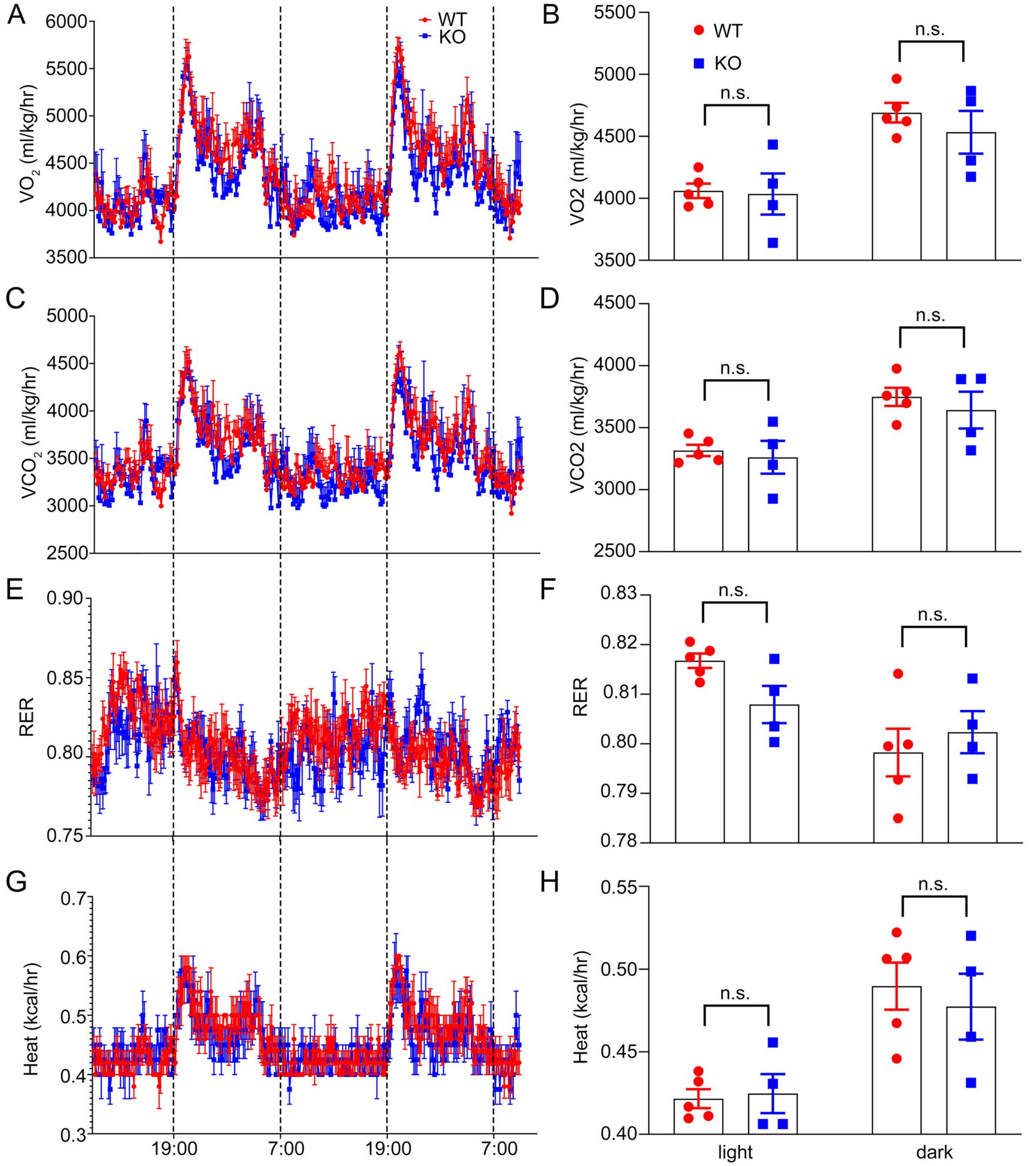

Figure S9. PRAP1 deficiency does not significantly affect energy expenditure of mice on a HFD. Energy expenditure of WT and PRAP1$^{-/-}$ mice on a HFD for 2 weeks were monitored using CLAMS for 48 hrs. (A, B) Oxygen consumption rate (VO2); (C,D) Carbon dioxide production rate (VCO2); (E,F) Respiratory exchange ratio VCO2/VO2 (RER); (G,H) Heat production rate. Data are expressed as mean ± SEM, n=4-5 for each genotype. The data shown in (B,D, F, H) are average of two dark and two light cycles. n.s., $p > 0.05$, un-paired Student's t-test.

Table S1. Primers used in RT-qPCR

**Gene**	**Locus**	**Primer**	**Sequence**	**Size** (bp)
apoB	NM_009693.2	Forward	CCAGACAACCTCTTCCTAAAGACT	70
		Reverse	TCAATGTTTATTTTGTTCCTGTTCA	
Prap1	NM_009475.2	Forward	AGGAAAAGCGTCCAGATGCC	126
		Reverse	TGGACATCGTCAGACATGGG	
Actb	NM_007393.5	Forward	AGTGTGACGTTGACATCCGT	100
		Reverse	TGCTAGGAGCCAGAGCAGTA	

Table S2. Antibodies used in this study

Antibodies	Sources	Identifier
MTP Antibody (8) (WB, IP)	Santa cruz	Cat# sc-135994
MTP Antibody (N-17) (IF)	Santa cruz	Cat# sc-33116
apoA-I (2G4) (WB)	Santa cruz	Cat# sc-69755
Albumin (F-10) (WB)	Santa cruz	Cat# sc-271605
Anti-Apolipoprotein B antibody (WB)	Abcam	Cat# ab20737
Monoclonal Anti-flag M2 (WB,IP)	Sigma	Cat# F1804
PRAP1 Antibody	Current study	
